# First-principles study of mercaptoundecanoic acid molecule adsorption and gas molecule penetration onto silver surface: an insight for corrosion protection†

**DOI:** 10.1039/d3ra06040c

**Published:** 2023-10-25

**Authors:** Chung-Hyok Kim, Chol Ryu, Yong-Hak Ro, Song-Il O, Chol-Jun Yu

**Affiliations:** a Institute of Electronic Materials, High-Tech and Development Centre, Kim Il Sung University PO Box 76 Pyongyang Democratic People's Republic of Korea; b Computational Materials Design (CMD), Faculty of Materials Science, Kim Il Sung University PO Box 76 Pyongyang Democratic People's Republic of Korea cj.yu@ryongnamsan.edu.kp; c Physics Department, O Jung Hub Chongjin University of Education Chongjin Hamgyong North Province Democratic People's Republic of Korea

## Abstract

Recently, 11-mercaptoundecanoic acid (MUA) molecule has attracted attention as a promising passivation agent of Ag nanowire (NW) network electrode for corrosion inhibition, but the underneath mechanism has not been elaborated. In this work, we investigate adsorption of MUA molecule on Ag(1 0 0) and Ag(1 1 1) surface, adsorption of air gas molecules of H_2_O, H_2_S and O_2_ on MUA molecular end surface, and their penetrations into the Ag surface using the first-principles calculations. Our calculations reveal that the MUA molecule is strongly bound to the Ag surface with the binding energies ranging from −0.47 to −2.06 eV and the Ag–S bond lengths of 2.68–2.97 Å by Lewis acid–base reaction. Furthermore, we find attractive interactions between the gas molecules and the MUA@Ag complexes upon their adsorptions and calculate activation barriers for their migrations from the outermost end of the complexes to the top of Ag surface. It is found that the penetrations of H_2_O and H_2_S are more difficult than the O_2_ penetration due to their higher activation barriers, while the O_2_ penetration is still difficult, confirming the corrosion protection of Ag NW network by adsorbing the uniform monolayer of MUA. With these findings, this work can contribute to finding a better passivation agent in the strategy of corrosion protection of Ag NW network electrode.

## Abstract 2

First-principles simulations of adsorption of MUA molecule on Ag(1 0 0) and Ag(1 1 1) surfaces, adsorption of H_2_O, H_2_S and O_2_ molecules and their penetrations onto the Ag surface were performed to elaborate the mechanism of corrosion protection.

## Graphical abstract

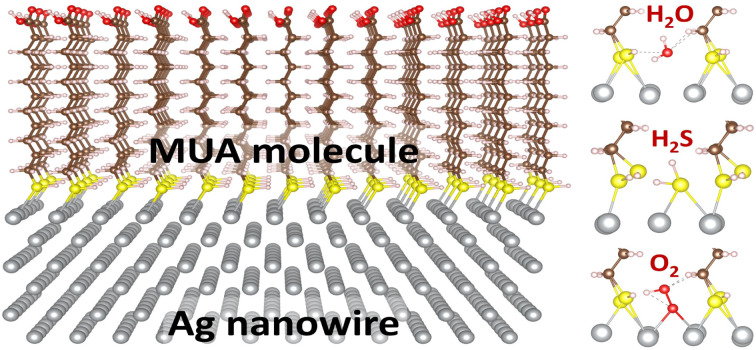

## 1 Introduction

Silver nanowire (NW) networks have attracted significant research attention in numerous applications including transparent flexible conductors, strain sensors, light emitting diodes (LEDs), liquids crystal displays (LCD), self-healing electronic devices and solar cells.^1–5^ This is due to their superior material properties such as high mechanical flexibility and high electrical conductivity combined with reasonable transparency.^6,7^ In particular, Ag NW networks show a great potentiality of promising transparent electrodes as an alternative to the films of metal oxides such as indium tin oxide (ITO).^8–10^ Although commercially wide use during the past decades, ITO films have problems of limited mechanical flexibility and high production cost owing to requiring high temperature and vacuum condition for deposition. On the contrary, Ag NWs can be mass produced at low cost through solution synthesis^11–13^ and their deposition on the surface can be easily realized by using the roll-to-roll processes at room temperature.^14,15^

In spite of such indisputable merits, Ag NW films have suffered from a critical problem of short-term chemical stability upon exposure to humidity or light.^16–20^ When exposed to air, silver is liable to oxidation or sulfidation, leading to a degradation of its electrical performance.^21–23^ Such silver corrosion is caused by chemical reactions occurring on the Ag surface with water (H_2_O) or hydrogen sulfide (H_2_S) and carbonyl sulfide (COS), which exist in air. From the experimental analyses,^19,22^ it was revealed that the silver sulfide (AgS) in the form of nanoparticles or discontinuous shells was created on the Ag NW surfaces. These kinds of artifact formed by corrosion cause a significant increase in electrical resistance of Ag NW electrodes. For instance, Deignan *et al.*^19^ found that the electrodes prepared from poly(vinylpyrrolidone) (PVP)-stabilized silver network (AgNW@PVP) became non-conductive after only a few weeks.

To address the issue, various approaches have been developed. Among them, it is the most general and widely used to coat Ag NW networks with thin passivation layers for protection from external actions. However, it is not easy to find a suitable protective layer for many applications due to the rigorous requirements such as being mechanically flexible, optically transparent, and inexpensive. For the applications of solar cells and LEDs, moreover, the current flow should be allowed through the NW passivation layer between the electrodes and devices. In recent years, there have been developed several kinds of passivation materials satisfying the above terms for Ag NW transparent electrodes.^1,24–26^ In particular, organic short molecules have been found to show many advantages over other materials. In fact, the strong binding between ligands and Ag atoms can enhance the chemical stability, while the transparency, conductivity and flexibility of the Ag electrodes are little damaged.^27–29^ Idier *et al.*^27^ demonstrated the higher chemical stability of Ag NW electrode passivated with triphenylphosphine (PPh_3_) (AgNW@PPh_3_) than AgNW@PVP electrodes. However, the AgNW@PPh_3_ electrodes exhibited a 500% increase in resistance after 110 days.^27^ The organothiols, such as 2-mercaptobenzimidazole (MBI), were also tested for passivation of Ag NW, finding that the resistance increase was only 67% after 120 days but the test was performed in a chamber without exposure to light.^29^

In this regard, the short molecule of 11-mercaptoundecanoic acid (MUA) has been widely used to passivate Ag or Au surfaces as it can be readily bound to the Ag or Au atoms through the thiolate bond.^30–35^ Through Raman spectroscopy analysis, Madeira it *et al.*^30^ demonstrated that the MUA molecules can easily replace the PVP molecules remaining on Ag NW networks. It was revealed that the MUA monolayer on Ag surface could effectively prevent the Ag corrosion by forming a packing order owing to the van der Waals (vdW) and electrostatic interactions between the alkyl chains.^36^ Furthermore, the formation of extra Ag_2_S artifacts has been proved to be avoided by saturating the Ag NW surface with S atoms and bonding of every Ag atoms to the S atoms of the MUA molecules. First-principles study based on the density functional theory (DFT) is of importance in exploring the adsorption and explaining the corrosion inhibition mechanism.^37–42^ Although some works for Ag NW itself^43,44^ and for PVP binding to Ag surface^36^ have been reported, first-principles works are little carried out for MUA@Ag NW complexes, remaining the understanding of corrosion protection mechanism indistinct.

In this work, we investigate adsorption of MUA molecules on Ag NW surface by using first-principles calculations within the DFT framework. For modeling of Ag surfaces, we choose the low index (1 0 0) and (1 1 1) surfaces and use the slab models with sufficient number of atomic layers and vacuum thickness. We determine the surface formation energies of the Ag surfaces from the bulk and the adsorption energies of MUA molecule on the surfaces. To get an insight into corrosion protection, we further simulate migrations of gas molecules in air such as H_2_O, O_2_ and H_2_S through the MUA layers with calculations of the corresponding activation barriers. The frontier molecular orbitals of MUA and charge transfer upon adsorption are analyzed to help understand the physicochemical nature.

## 2 Computational methods

The DFT calculations were carried out using the pseudopotential and pseudo atomic orbital (PAO) method as implemented in the SIESTA package (version 4.1.b3).^45^ The electrostatic interaction between the valence electrons and ionic cores was described using the soft norm-conserving pseudopotentials in the Troullier–Martins type,^46^ which were constructed by using the ATOM code included in the package with the valence electron configurations of atoms like Ag-5s^1^5p^0^4d^10^4f^0^, S-3s^2^3p^4^3d^0^4f^0^, C-2s^2^2p^2^3d^0^4f^0^, O-2s^2^2p^4^3d^0^4f^0^, and H-1s^1^2p^0^3d^0^4f^0^. In constructing the pseudopotential, we considered the empty states up to *l*_max_ = 3 (f state) by applying the generalized approach,^47^ and used the cutoff radii of 1.49, 1.54, 1.14, and 2.15 Bohr for s, p, d, and f states, respectively. The exchange-correlation interaction between the valence electrons was described by using the Perdew–Burke–Ernzerhof (PBE) functional^48^ within the generalized gradient approximation (GGA). The dispersive van der Waals (vdW) interactions between the molecules and surfaces were included by using the semi-empirical Grimme's approach^49^ with the proper parameters provided in the package.

For PAO basis sets, we employed split-valence double-*ζ* plus polarization (DZP) sets for all the atoms with an energy shift of 50 meV and a split norm of 0.25. The cutoff energy for setting the wavelength of the plane waves was set to be 300 Ry, which yielded a real spacing between the grid points of 0.07 Å for wave functions and electron density. For the Brillouin zone integration, the *k*-point mesh of (8 × 8 × 1) was used in surfaces and molecule adsorption on the surface, while only Γ point was used in the calculation of isolated molecule. In the structural optimization, the atoms were relaxed until the atomic forces converged to 0.02 eV Å^−1^. The activation energies for migration of gas molecules were evaluated by applying the climbing-image nudged elastic band (NEB) method,^50^ as implemented in the Python script of Pastafarian in connection with the SIESTA program as applied in our previous work.^51,52^ We used 39 NEB images to discretize the path while allowing the atomic relaxations with the force convergence threshold of 0.02 eV Å^−1^. We checked that these computational settings for the PAO basis sets and the real spacing grid provided well converged results, as already proved in the previous works.^53–56^

The low index Ag(1 0 0) and (1 1 1) surfaces were chosen because these surfaces were found to be the most stable and thus form the facets of Ag NW.^11,36^ Different slab models were constructed for the Ag(1 0 0) and Ag(1 1 1) surfaces with different surface cells and different number of atomic layers. We tested (3 × 3) and (2 × 2) surface cells with increasing number atomic layers up to 9 and 10 for the Ag(1 0 0) and Ag(1 1 1) surfaces, respectively. The vacuum thickness in the 3-dimensional periodic supercell was set to be 35 Å along the *z* axis, which is long enough to eliminate the artificial interaction between the periodic images. The upper three layers (on both surfaces of slab) were allowed to relax, while the remaining center layers were fixed at their bulk positions. After the surface relaxation, the surface formation energy was calculated as follows,1
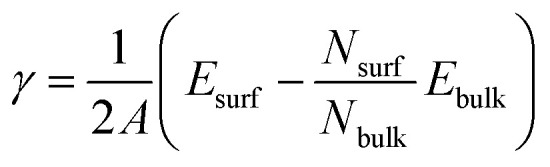
where *A* is the area of the surface cell, *N*_surf_ and *N*_bulk_ are the numbers of atoms in the surface supercell and the bulk unit cell, and *E*_surf_ and *E*_bulk_ are the corresponding total energies. Through the convergence test for the formation energies of Ag(1 0 0) and (1 1 1) surfaces, we confirmed that the present settings of atomic layer and vacuum thickness provided the accuracy of *γ* as 0.01 J m^−2^ (see Fig. S1, ESI†).

An isolated MUA molecule within a big cubic supercell with a lattice constant of 35 Å was optimized, and adsorbed on the Ag(1 0 0) and Ag(1 1 1) surfaces with different monolayer (ML) coverages. For configuration of MUA adsorption on the Ag surfaces, the molecule was forced to be adsorbed in the way of its chain orienting vertically to the surface with the contact between the S–H end of MUA and the surface Ag atoms in reference to the experimental findings.^30^ To estimate the binding strength between the adsorbed MUA molecule and the Ag surface, we calculated the binding energies as follows,

2 *E*_b_ = *E*_mol+surf_ − (*E*_mol_ + *E*_surf_)

where *E*_mol+surf_, *E*_mol_, and *E*_surf_ are the total energies of the supercells for the MUA molecule-adsorbed Ag surface, isolated MUA molecule, and pristine Ag surface, respectively. With this definition, negative binding energies indicate attraction while positive values indicate repulsion between the MUA molecule and the Ag surface. To check whether the MUA monolayer formed on the Ag surface can protect the corrosion in the air, we simulated the migrations of gas molecules included in the air, such as O_2_, H_2_O and H_2_S, from the top of MUA molecule to the Ag surface, and calculated the corresponding activation barriers by applying the NEB method.

## 3 Results and discussion

**Fig. 1:**
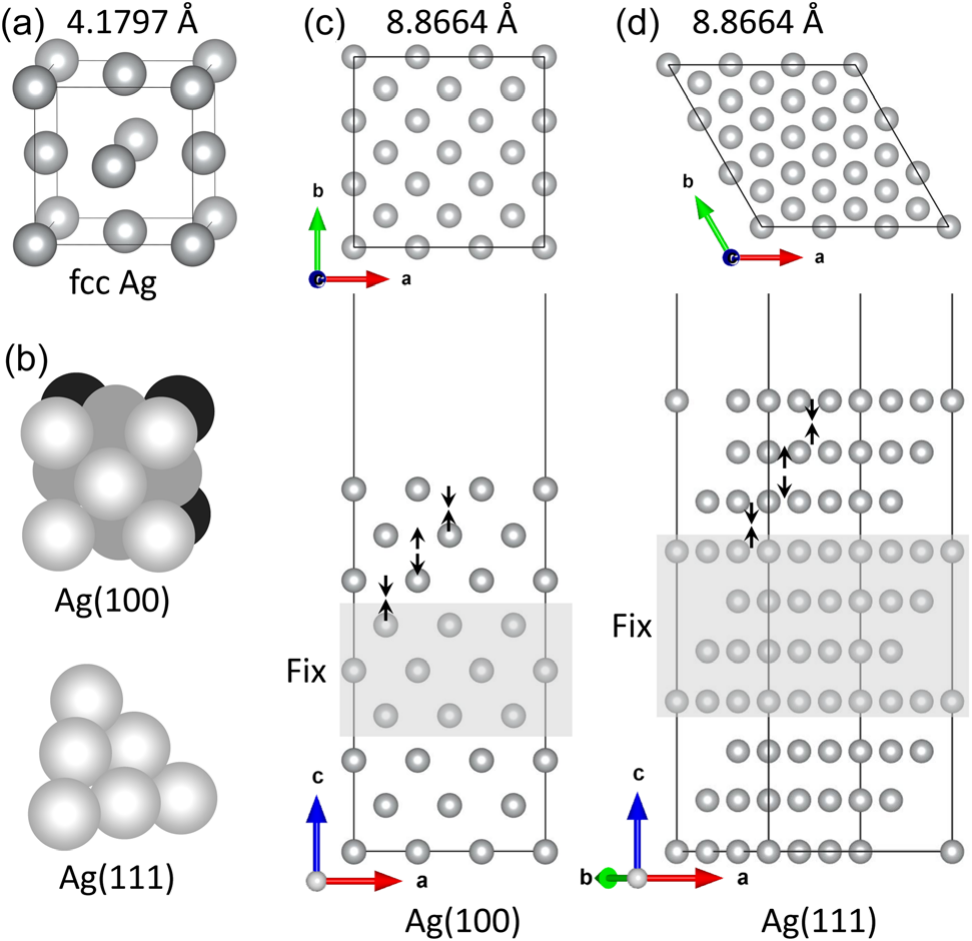
(a) Unit cell of Ag crystal in fcc phase with an optimized lattice constant (4.1797 Å), (b) atomic packing of the Ag(1 0 0) and Ag(1 1 1) surfaces, and slab supercells of (c) Ag(1 0 0) and (d) Ag(1 1 1) surfaces with (3 × 3) surface cells (lattice constant 8.8664 Å). The upper three layers on both surfaces of slab are relaxed and the central layers (gray-colored region) are fixed at their bulk positions. The arrows indicate the layer relaxation way.

First, we optimized the unit cell of Ag crystal in face-centered cubic (fcc) phase and determined the lattice constant to be 4.180 Å with a slight overestimation of 2.2% compared to the experimental value of 4.088 Å^11^ (see Fig. 1(a)). This overestimation agreed with the general trend of PBE-GGA exchange-correlation functional for metals and the previous DFT calculation results.^57–62^ The cohesive energy, *E*_c_ = *E*_fcc_ − *E*_a_ where *E*_fcc_ and *E*_a_ are the total energies of fcc-Ag unit cell per atom and the isolated Ag atom, was calculated to be −2.58 eV, which is comparable with the experimental value of −2.96 eV and the previous DFT value of −2.52 eV.^62^

Then, the surface formation energies (*γ*) of the pristine Ag(1 0 0) and Ag(1 1 1) surfaces were determined after completing the surface relaxations. As listed in Table 1, the *γ* values were determined to be 1.11 and 0.89 J m^−2^ (or 0.55 and 0.46 eV per atom) for Ag(1 0 0) and Ag(1 1 1) surfaces, respectively. These agreed reasonably with the previous DFT result of 0.78 J m^−2^ (ref. 60) (0.55 and 0.36 eV per atom^62^) and the experimental result of 1.27 J m^−2^ (ref. 63) (0.88 and 0.55 eV per atom^64^) for Ag(1 0 0) surface (Ag(1 0 0) and Ag(1 1 1) surfaces, respectively). In Table 1, the inter-layer relaxation for the uppermost four layers (Δ*d*_12_, Δ*d*_23_, Δ*d*_34_) are given as a percentage of the fixed bulk interlayer distance. The distance between the first and second layers (Δ*d*_12_) contract 2.5 and 2.6% in good agreement with the experimental values of 0.0 ± 1.5%^65^ and 2.5%^66^ for Ag(1 0 0) and Ag(1 1 1) surfaces, respectively. For the second-third interlayer distance (Δ*d*_23_), we found slight expansions of 0.1 and 0.7%, which agreed well with the experimental results of 0.0 ± 1.5%^65^ and 0.6%^66^ for the (1 0 0) and (1 1 1) surfaces, respectively. Then, the third-fourth interlayer Δ*d*_34_ was found to be contracted by 0.8 and 0.1% for the (1 0 0) and (1 1 1) surfaces, respectively. For Ag bulk and pristine Ag surfaces, our calculations can be said to be reliable when compared with the previous DFT works and the experimental results.

**Table 1:** Surface formation energy (*γ*) and inter-layer relaxation (Δ*d*) for pristine Ag(1 0 0) and Ag(1 1 1) surfaces in comparison with the previous (Prev.) DFT and experimental (Exp.) results

	Ag(100)			Ag(111)		
	This	Prev.	Exp.	This	Prev.^*c*^	Exp.
*γ* (J m^−2^)	1.11	0.78^a^	1.27^b^	0.89	0.76	
*γ* (eV per atom)	0.55	0.55^c^	0.88^d^	0.46	0.36	0.55^d^
Δ*d*_12_ (%)	−2.46	−1.87^c^	0.0 ± 1.5^e^	−2.63	−0.30	−2.50^f^
Δ*d*_23_ (%)	0.10	0.51^c^	0.0 ± 1.5^e^	0.71	0.04	0.60^f^
Δ*d*_34_ (%)	−0.80	0.30^c^		−0.10	0.16	

a Ref. 60.

b Ref. 63.

c Ref. 62.

d Ref. 64.

e Ref. 65.

f Ref. 66.

At the next step, we performed the atomic relaxations of isolated MUA molecule placed in the cubic supercell with a lattice constant of 35 Å. Before doing that, a conformation search was carried out by applying the stochastic search approach with the Conformer module in the Materials Studio package. As a result, the lowest energy conformation with the linear zig-zag C chain was derived. After optimization, the average bond lengths were measured to be 1.523 Å for C–C, 1.826 Å for C–S, 1.373 Å for C–O (1.217 Å for C–OH), 1.097 Å for C–H, 1.353 Å for S–H, and 0.990 Å for O–H, respectively. These bond lengths are reasonable in reference to the general knowledge of bond lengths. The typical bond angles were also measured to 93.48° for H–S–C, 115.05° for S–C–C, 115.58° for C–C–C, 105.71° for H–C–H, 122.45° for O–C–O, and 113.04° for C–C–O (124.51° for C–C–OH) (see Table S1, ESI†).

**Fig. 2:**
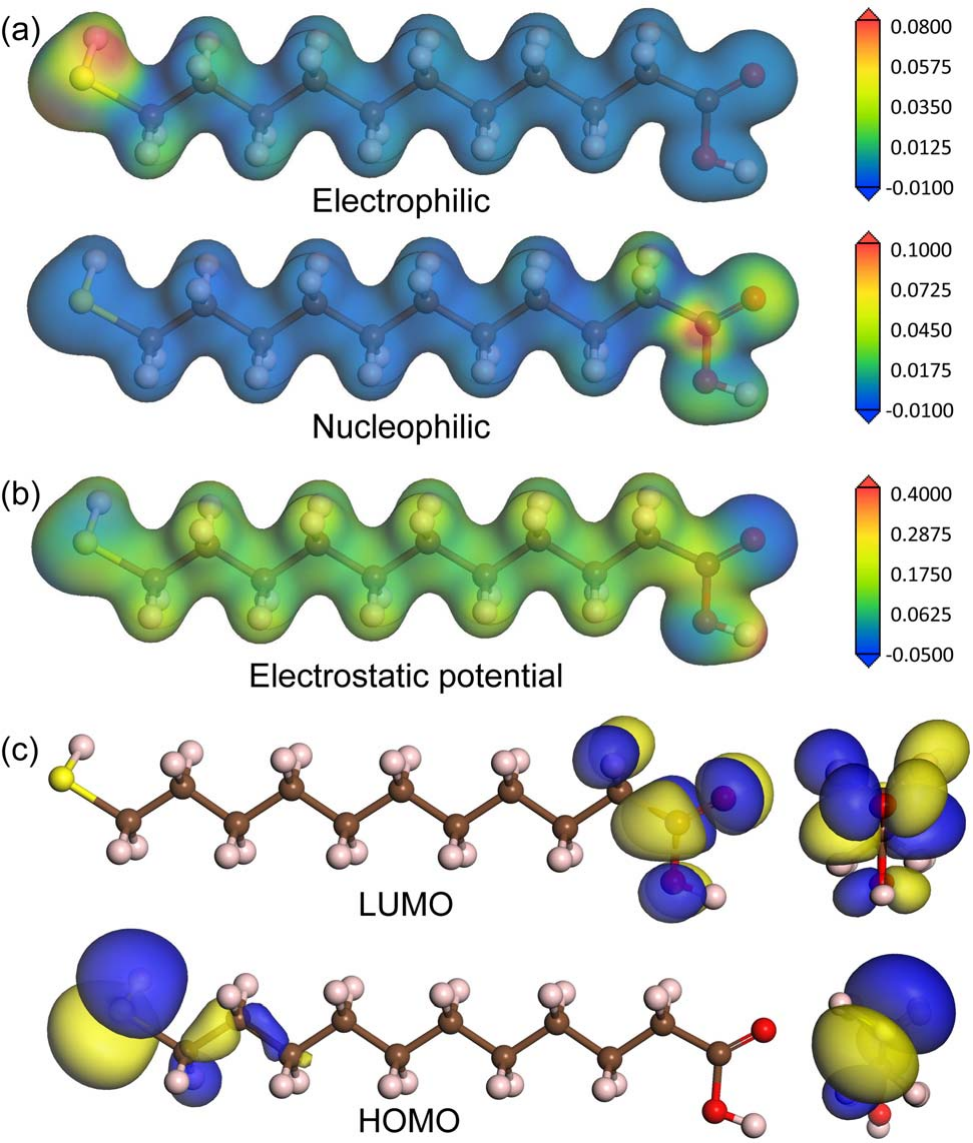
(a) Isodensity surfaces of Fukui functions representing the reactivity with respect to electrophilic (top) and nucleophilic (bottom) attacks and of (b) electrostatic potential, mapped on isosurface of total electron density at the value of 0.2|*e*| Å^−3^. (c) Isosurface view of frontier molecular orbitals including LUMO and HOMO in MUA molecule. Brown-, yellow-, red-, and pink-colored balls represent C, S, O, and H atoms, respectively.

To estimate the chemical reactivity of species of MUA molecule, we calculated the Fukui function, electrostatic potential and frontier molecular orbitals, as shown in Fig. 2. The local reactivity of a molecule can be qualitatively described by measuring the sensitivity of the charge density with respect to the loss or gain of electrons, *i.e.*, Fukui function. According to the frontier orbital theory of Fukui, an electrophile accepts a pair of electrons like a Lewis acid to form a new covalent bond, whereas a nucleophile provides a pair of electrons like a Lewis base. Fig. 2(a) shows the isodensity surfaces of Fukui functions mapped on the isosurface of total electron density at the value of 0.2|*e*| Å^−3^ to represent the local reactivity with respect to the electrophilic and nucleophilic attacks, clearly indicating that the sulfur atom is the electrophile while the oxygen atoms are the nucleophiles. In fact, the Fukui indices for electrophilic attack with were found to be 0.595, −0.015, 0.026 and 0.003 for S, C, H and O atoms, and those for nucleophilic attack were to be 0.014, 0.007, 0.023 and 0.175 for S, C, H and O atoms. We also show the isodensity surface of electrostatic potential mapped on the total electron density in Fig. 2(b), confirming that the sulfur and oxygen atoms are the reacting species with negative values of electrostatic potential. In addition, the highest occupied molecular orbital (HOMO) and the lowest unoccupied molecular orbital (LUMO) were found around the sulfur and oxygen atoms with the energy levels of −6.0 and −1.1 eV (HOMO–LUMO gap of 4.9 eV), respectively, as shown in Fig. 2(c). From these findings, we can conclude that the sulfur atom of MUA molecule can react with Ag atoms on top of surface through Lewis acid–base reaction.

Then, we considered adsorption of MUA molecule on Ag surface to elucidate the mechanism of passivation strategy of Ag NW network with MUA. In the experiment, it was found that immersing the Ag NW networks in a solution of MUA resulted in a uniform thin layer of MUA on the Ag NWs.^30^ Therefore, we constructed the initial configuration of MUA adsorption on Ag surface by arranging MUA molecule backbone perpendicular to the surface while making the sulfur atom contacting with the Ag atoms. Different adsorbate monolayer coverages were considered, such as one molecule on (3 × 3) surface cell (0.11 ML), one molecule on (2 × 2) cell (0.25 ML) and two molecules on (2 × 2) cell (0.5 ML). With respect to the adsorption position, we tested 12 different configurations to select one configuration with the lowest total energy (see Fig. S2–S5, ESI†).

**Fig. 3:**
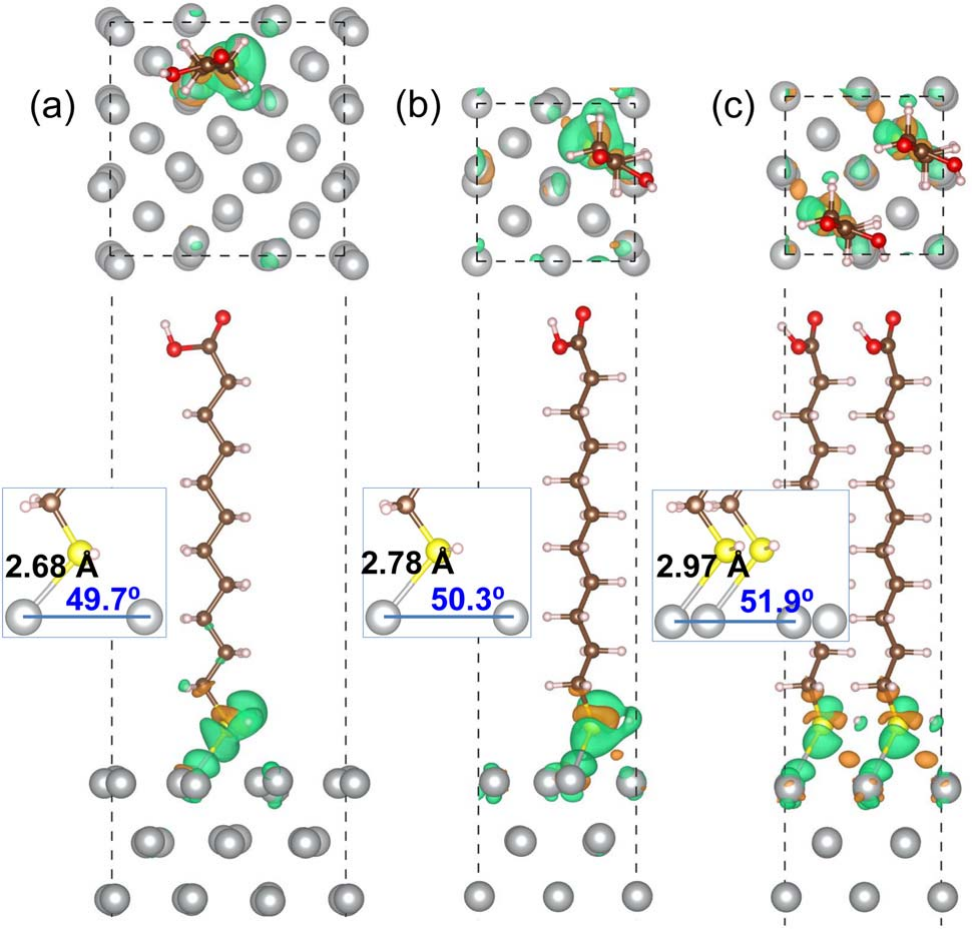
Top and side view of optimized structures of MUA molecule adsorbed on Ag(1 0 0) surface with (a) (3 × 3) cell and (b) (2 × 2) cell, and (c) two MUA molecules on (2 × 2) cell. Isosurface of electron density difference at the value of 0.015|*e*| Å^−3^ is also shown, where orange (green) color represents electron accumulation (depletion). Insets show the Ag–S bond length (Å unit) and the S–Ag–Ag bond angle (deg unit) formed on the Ag(1 0 0) surface.

Fig. 3 shows the optimized geometries of MUA@Ag(1 0 0) complexes with the three different coverages. It was found that the S atom of MUA molecule formed a new covalent or ionic bond with the Ag atom on the top surface by adsorption in the three complexes. In fact, from the isosurface view of electron density difference, calculated by Δ*ρ*(**r**) = *ρ*_mol+surf_(**r**) − [*ρ*_mol_(**r**) + *ρ*_surf_(**r**)], the sulfur atom was found to gain electrons (that is, Lewis acid), whereas the silver atoms lost electrons (Lewis base). This agrees well with the conclusion derived from the above analysis of Fukui function of MUA molecule. As the geometrical characteristics, therefore, the Ag–S bond length and S–Ag–Ag bond angle were measured. With increasing the coverage, they were found to gradually increase; 2.68 Å, 49.7° for 0.11 ML, 2.78 Å, 50.3° for 0.25 ML, and 2.97 Å, 51.9° for 0.5 ML coverages, as shown in the insets of Fig. 3 and listed in Table 2. This indicates that the interaction between MUA molecule and Ag surface through sharing (or transferring) a pair of electrons is enhanced as increasing the concentration of adsorbate molecule. The reason might be an enhancement of interaction between molecules when increasing the concentration. To quantitatively assess the binding strength, we calculated the binding energy *E*_b_ using eqn (2) and presented the results in Table 2. The binding energies were obtained to be negative, indicating the attraction between MUA and Ag surface, and to decrease in magnitude from −1.62, −0.77 to −0.47 eV as increasing the coverage. It is worthy noting that the PBE functional itself without vdW gives slightly larger bond length *d*_Ag–S_ and bond angle *θ*_S–Ag–Ag_ and smaller binding energy *E*_b_ when compared with those by PBE + vdW, as shown in Table 2.

**Table 2:** Ag–S bond length (*d*_Ag–S_), S–Ag–Ag bond angle (*θ*_S–Ag–Ag_), and binding energy (*E*_b_) in MUA-adsorbed Ag(1 0 0) and Ag(1 1 1) surface complexes with difference adsorbate coverage values. Values in parenthesis are obtained by PBE only without vdW

Surface	Coverage (ML)	*d*_Ag–S_ (Å)	*θ*_S–Ag–Ag_ (deg)	*E*_b_ (eV)
Ag(1 0 0)	0.11	2.68 (2.75)	49.7 (60.2)	−1.62 (−1.35)
	0.25	2.78 (2.84)	50.3 (60.6)	−0.77 (−0.58)
	0.50	2.97 (3.06)	51.9 (61.7)	−0.47 (−0.31)
Ag(1 1 1)	0.11	2.68 (2.77)	61.2 (68.7)	−2.06 (−1.79)
	0.25	2.70 (2.79)	61.8 (69.1)	−0.91 (−0.64)
	0.50	2.71 (2.81)	62.9 (70.5)	−0.52 (−0.23)

**Fig. 4:**
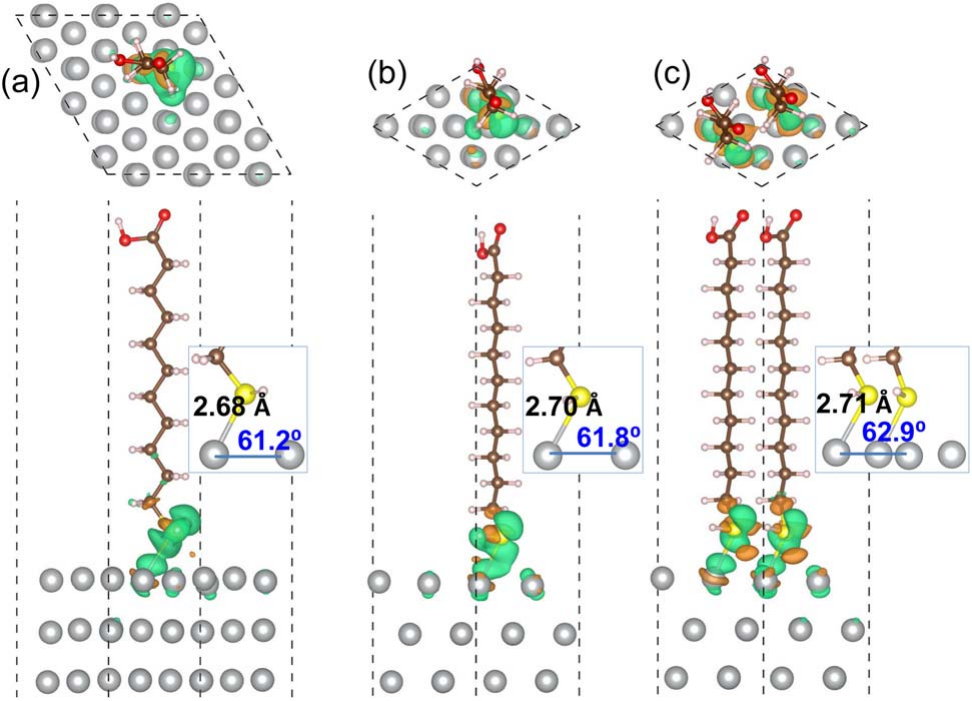
Top and side view of optimized structures of MUA molecule adsorbed on Ag(1 1 1) surface with (a) (3 × 3) cell and (b) (2 × 2) cell, and (c) two MUA molecules on (2 × 2) cell. Isosurface view of electron density difference is also shown at the value of 0.015|*e*| Å^−3^, where orange (gree) color represents electron accumulation (depletion). Insets show the Ag–S bond length (Å unit) and the S–Ag–Ag bond angle (deg unit) formed on the Ag(1 1 1) surface.

For the MUA adsorption on the Ag(1 1 1) surface, similar results were obtained. As shown in Fig. 4, however, the increase of Ag–S bond length was almost negligible as from 2.68, 2.70 to 2.71 Å with the increase of adsorbate concentration. This indicates that the attraction between MUA and Ag(1 1 1) surface is clearly stronger than that with the Ag(1 0 0) surface, although the degree of S–Ag–Ag bond angle change was similar to the cases of Ag(1 0 0) surface. The binding energies *E*_b_ with the Ag(1 1 1) surface were also larger in magnitude than those with the Ag(1 0 0) surface at the same coverage. It is worth noting that the binding energies are still negative as −0.47 and −0.52 eV for the Ag(1 0 0) and (1 1 1) surface at 0.5 ML coverage, which might be the largest value of coverage in reference to the lateral size of MUA molecule. Therefore, the adsorbate complexes of two MUA molecules on (2 × 2) surface cell will be used for further consideration in the following.

**Fig. 5:**
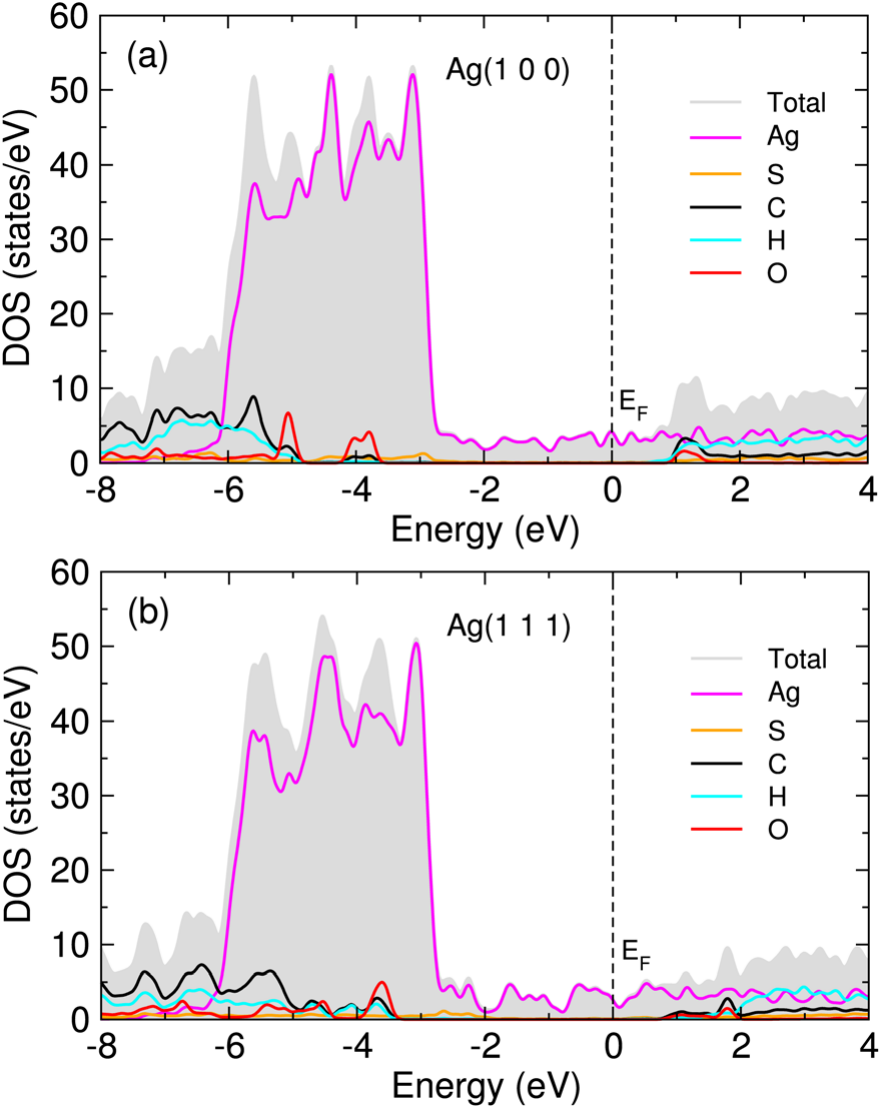
Atom-projected density of states (DOS) for 2 MUA adsorbed (a) Ag(1 0 0) and (b) Ag(1 1 1) (2 × 2) surface supercell complexes. Fermi energy (*E*_F_) is set to zero.

In order to get an insight into adsorption, we calculated the atom-projected density of states (DOS) for the MUA adsorbed Ag surface complexes. Fig. 5 shows the calculated DOS for two MUA adsorbed Ag(1 0 0) and Ag(1 1 1) (2 × 2) surface complexes (see Fig. S6 for one MUA adsorbed Ag surface complexes, ESI†). The frontier molecular orbitals of MUA bracket the Fermi energy, where the LUMO of MUA is ∼1 eV above *E*_F_ and the HOMO of MUA is 3−4 eV below the Fermi level, being similar to the case of PVP adsorbed Ag surface complexes.^36^ The occupied molecular orbitals of MUA are mainly composed of sulfur and oxygen, while the unoccupied molecular orbitals are originated from C, H and O atoms. For the occupied orbitals of MUA, we see the clear overlap between the p orbitals of MUA (those of S and O) and the d states of Ag, indicating their hybridization and thus the formation of new chemical bonds.

When compared with the previous DFT works for PVP adsorption on Ag(1 0 0) and (1 1 1) surfaces,^36^ our calculation values of surface bond length *d*_Ag–S_ are similar to the surface bond length *d*_Ag–O_ for PVP cases of 2.66 and 2.70 Å for (1 0 0) and (1 1 1) surfaces, respectively. Meanwhile, the *E*_b_ values at 0.5 ML are slightly smaller than those for PVP cases of −0.78 and −0.69 eV for (1 0 0) and (1 1 1) surfaces, respectively. This indicates that the attraction of Ag surface with MUA molecule is slightly weaker than that with PVP molecule, possibly due to the longer length of MUA molecule. For the adsorption of ethylene on the Ag(1 0 0) surface,^61^ the surface bond length (*d*_Ag–C_ = 2.82 Å) was larger and the binding energy (−0.10 eV) was smaller than our calculation values. Note that the previous calculations for ethylene were performed without consideration of vdW interaction. Anyhow, our calculations revealed that the uniform thin layer of MUA on Ag surface can be formed exothermically and thus the corrosion of Ag NW is expected to be effectively inhibited.

**Fig. 6:**
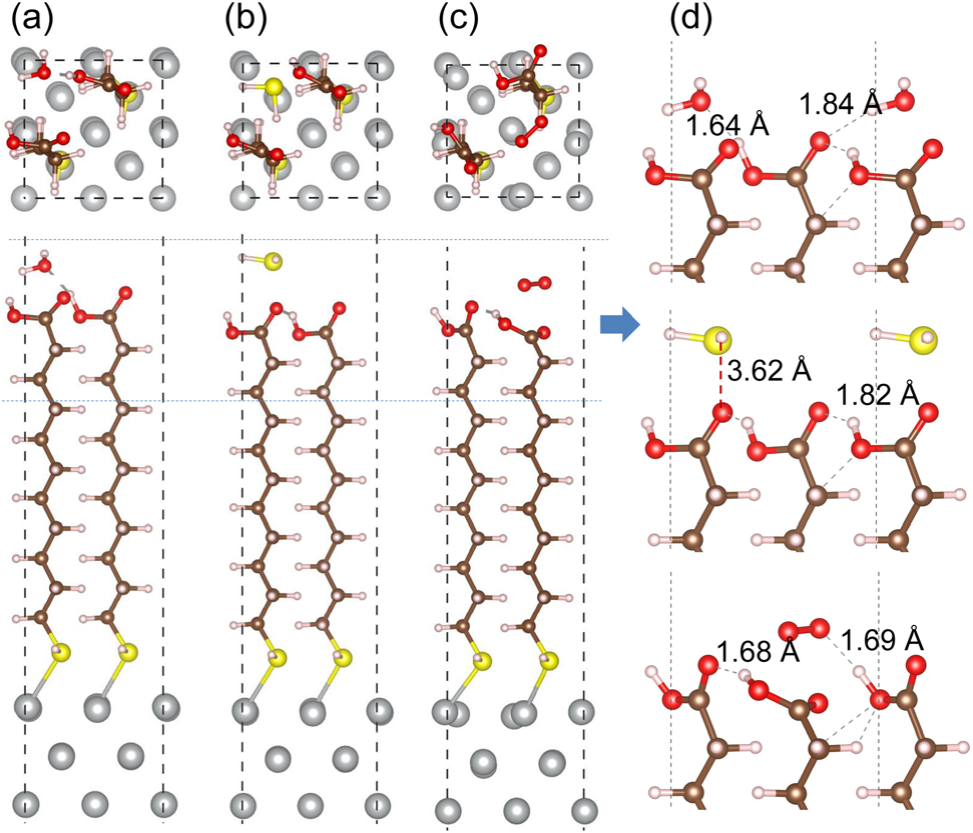
Top and side views of optimized structures of MUA@Ag(1 0 0) complexes with an adsorbed gas molecule of (a) H_2_O, (b) H_2_S and (c) O_2_. (d) Side view of enlarged adsorption region indicated by horizontal dotted lines in each complex with relevant bond lengths in angstrom unit.

In order to assess corrosion resistivity of MUA monolayer formed on Ag surface, we investigate the adsorption and migration of molecules included in the air, such as H_2_O, H_2_S and O_2_. It is reasonable that these molecules are expected to be adsorbed on top of MUA molecule (the end of carboxyl group) adsorbed on the Ag surface. Fig. 6 shows the optimized geometries of gas molecule-adsorbed MUA@Ag(1 0 0) complexes. For the case of H_2_O adsorption, we found the O⋯H hydrogen bonds with bond lengths of 1.64 and 1.84 Å formed between the H_2_O molecule and the O atoms of carboxyl group of MUA molecule. The similar O⋯H hydrogen bond length of 1.69 Å was found in the case of O_2_ adsorption. However, the H_2_S molecule was found to be away from the MUA molecule with a distance of 3.62 Å between S and O atoms. As a measure of binding strength, the binding energies were calculated as −1.21, −0.99, and −0.53 eV for H_2_O, O_2_, and H_2_S, respectively. The negative values of binding energy indicate the attraction between the gas molecule and the MUA@Ag(1 0 0) complex, and their magnitudes imply the order of binding strength as H_2_O → O_2_ → H_2_S in accordance with the bonding characteristics. To get an insight into selective protection of Ag surface by MUA, we also calculated the binding energies of these molecules to the bare Ag surface. The binding energies were −0.86, −0.80, and −2.76 eV for H_2_O, H_2_S, and O_2_, respectively. The binding energy for oxygen molecule is remarkably larger than those for H_2_O and H_2_S, since the former is chemisorption while the latters are physisorption on the Ag(1 0 0) surface (see Fig. S7, ESI†). This indicates that the Ag surface is prone to be corrodible by reaction with oxygen in air.

**Fig. 7:**
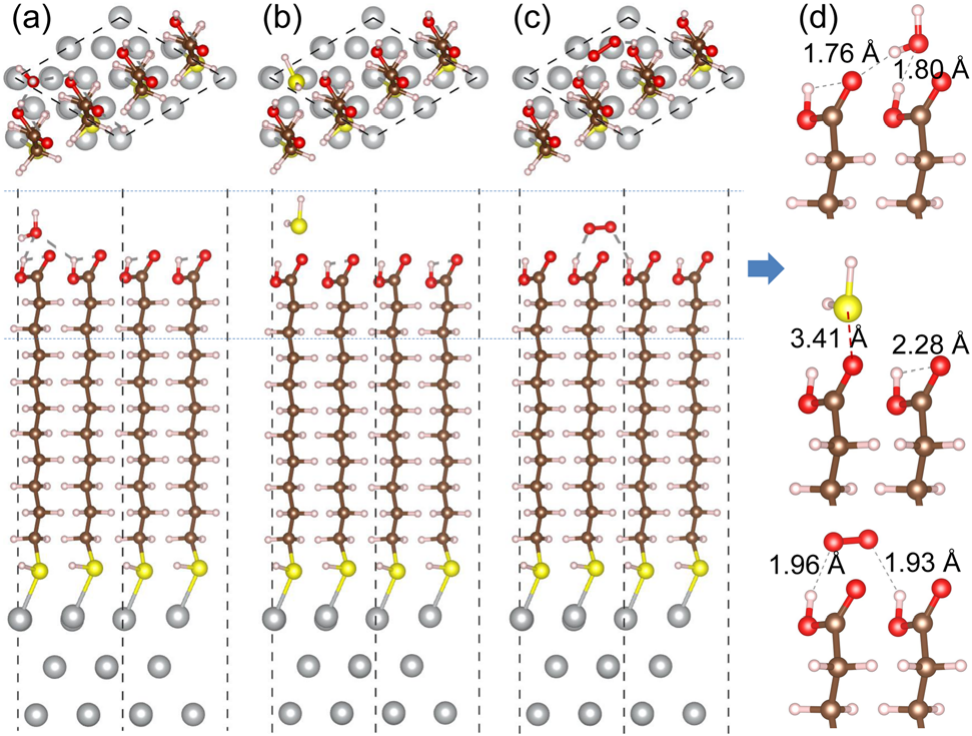
Top and side views of optimized structures of MUA@Ag(1 1 1) complexes with an adsorbed gas molecule of (a) H_2_O, (b) H_2_S and (c) O_2_. (d) Side view of enlarged adsorption region indicated by horizontal dotted lines in each complex with relevant bond lengths in angstrom unit.

For the cases of adsorption on MUA@Ag(1 1 1) complex, similar findings were obtained as shown in Fig. 7. The H_2_O molecule was found to be bound to MUA molecule *via* the O⋯H hydrogen bond with bond lengths of 1.76 and 1.80 Å and a binding energy of −1.08 eV. For the case of O_2_ adsorption, the hydrogen bond lengths (1.93, 1.96 Å) were more or less larger compared to the Ag(1 0 0) case and the binding energy was calculated to be −0.69 eV. When the H_2_S molecule was adsorbed, the distance between the S and O atoms was measured to be 3.41 Å and the binding energy was −0.75 eV. These adsorption complexes are regarded as the initial states for migration of gas molecule as will be considered below. The binding energies of H_2_O, H_2_S, and O_2_ to the bare Ag(1 1 1) surface were calculated to be −0.79, −0.94, and −2.20 eV, respectively (see Fig. S8, ESI†). Again, the Ag surface is prone to be oxidized in air.

We then proceeded with the investigation of migration of gas molecules of H_2_O, H_2_S and O_2_ in the air along the narrow and long path formed in the space surrounded by MUA molecules adsorbed on the Ag surface. Starting from the initial position considered above, the gas molecule was enforced to move to the top of Ag surface through the intermolecular space. Due to being relatively long, the migration path was divided into 6 sections with their own starting and end points as labeled from S1 to S7. By applying the NEB method, we determined the activation barrier for molecular migration in each section with clarifying the optimized geometries during the migration. In particular, the transition state (TS) was identified in each section, and the final state (FS) was analyzed in the last section.

**Fig. 8:**
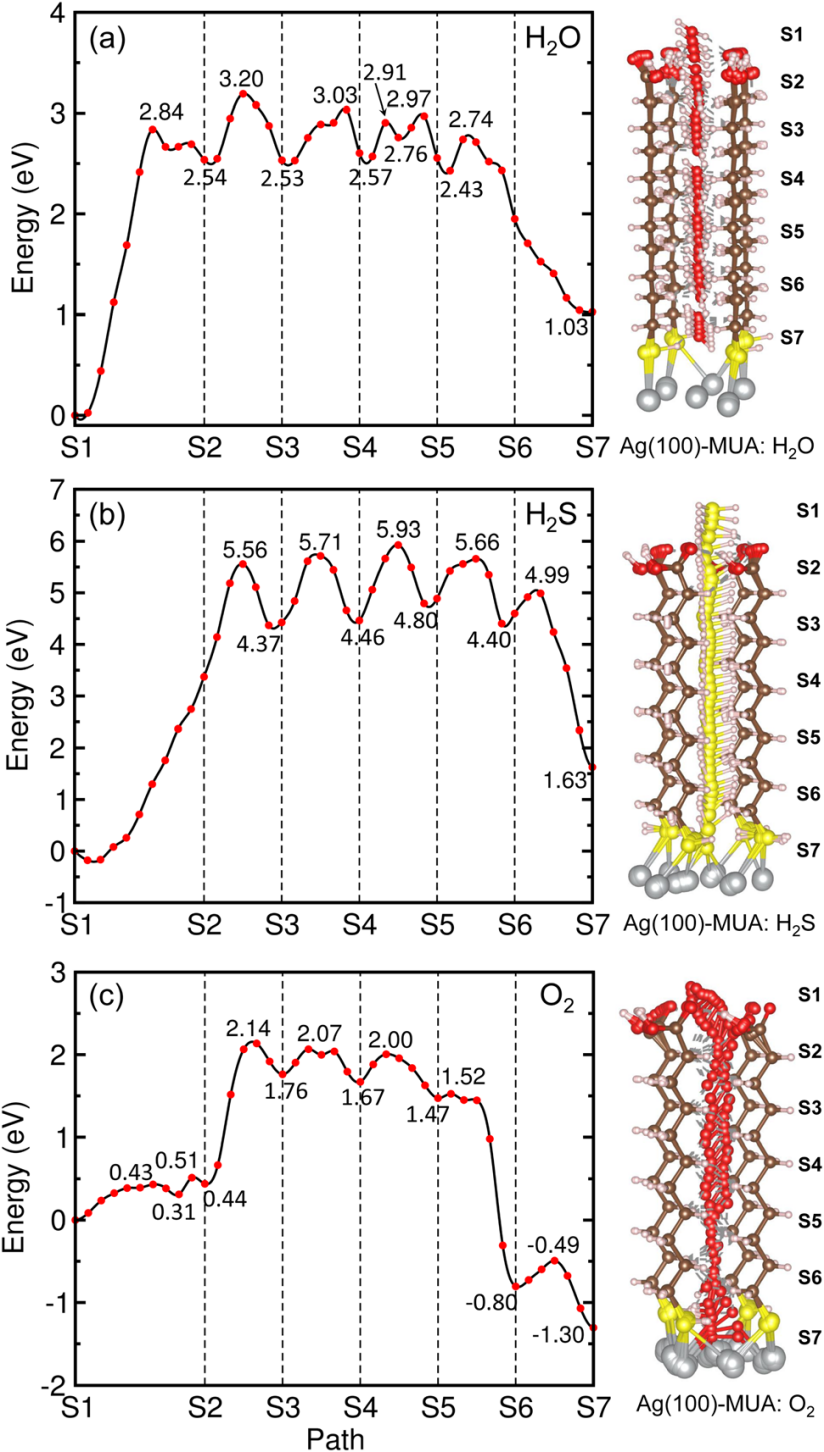
Energy profiles (left panel) for migrations of gas molecules of (a) H_2_O, (b) H_2_S and (c) O_2_ from top of MUA molecule to Ag(1 0 0) surface through space between adjacent MUA molecules, and the corresponding geometries during migration (right panel).

Fig. 8 depicts the energy profiles for migrations of the gas molecules of H_2_O, H_2_S and O_2_ with their optimized geometries in the MUA@Ag(1 0 0) complex. For the case of H_2_O migration (Fig. 8(a)), the migration barriers were 2.84, 0.66, 0.50, 0.34, 0.21, and 0.31 eV in the 6 sections determined by energy difference between the TS state and the local minimum state in each section. At the transition and final states, the hydrogen bonds were found between the H_2_O and MUA molecules (see Fig. S9, ESI†). The calculated activation barriers indicate that the insertion of H_2_O into MUA in the first section is the most difficult and then the migrations along the path of MUA backbone are relatively easier. At the last step of the migration, there is no barrier, indicating the spontaneous movement of the H_2_O molecule. In fact, the H_2_O molecule at the final state was found to be away from the Ag surface with a distance of 3.10 Å and thus not bound to the Ag atoms. For the case of H_2_S molecule (Fig. 8(b)), no barrier (no TS) was found in the first section, and then the barriers were 5.74, 1.34, 1.47, 0.86 and 0.59 eV in the following sections (see Fig. S10, ESI†). The high barrier for migration in the first and second sections implies that the carboxyl (–COOH) group of the MUA molecule hinders the insertion of H_2_S molecule more strongly than the H_2_O molecule. Although the barriers for the migration in the following sections are much lower than the first barrier, they are clearly higher than those for H_2_O migrations, indicating that the penetration of H_2_S molecule is more difficult. At the final state, the Ag–S bonds with bond lengths of 2.56 and 2.69 Å were newly formed with the penetrated H_2_S molecule, resulting in the formation of adsorbate complex of Ag_2_SH_2_.

As illustrated in Fig. 8(c), two transition states were found in the first section migration of O_2_ molecule with relatively low barriers of 0.43 and 0.20 eV. This indicates that the binding between the inserted O_2_ molecule and the carboxyl (–COOH) group of MUA is very weak compared with the H_2_O and H_2_S molecules. For the subsequent migration in the following sections, the barriers were found to be 1.70, 0.31, 0.33, 0.05 and 0.31 eV, which are comparable with those for the H_2_O migration (see Fig. S11, ESI†). At the final state, an adsorbate complex of Ag_2_O–Ag_2_O was formed with the average Ag–O bond length of 2.28 Å. It is worth noting that the final states are energetically higher than the initial states for H_2_O (1.03 eV) and H_2_S (1.63 eV), but it is lower for O_2_ (−1.30 eV). With the obtained highest barriers, it can be said that the H_2_S molecule (5.74 eV) is the most difficult to be penetrated into the Ag surface through the interstitial space, whereas the O_2_ molecule (1.70 eV) is the easiest and the H_2_O molecule (2.84 eV) is moderate.

**Fig. 9:**
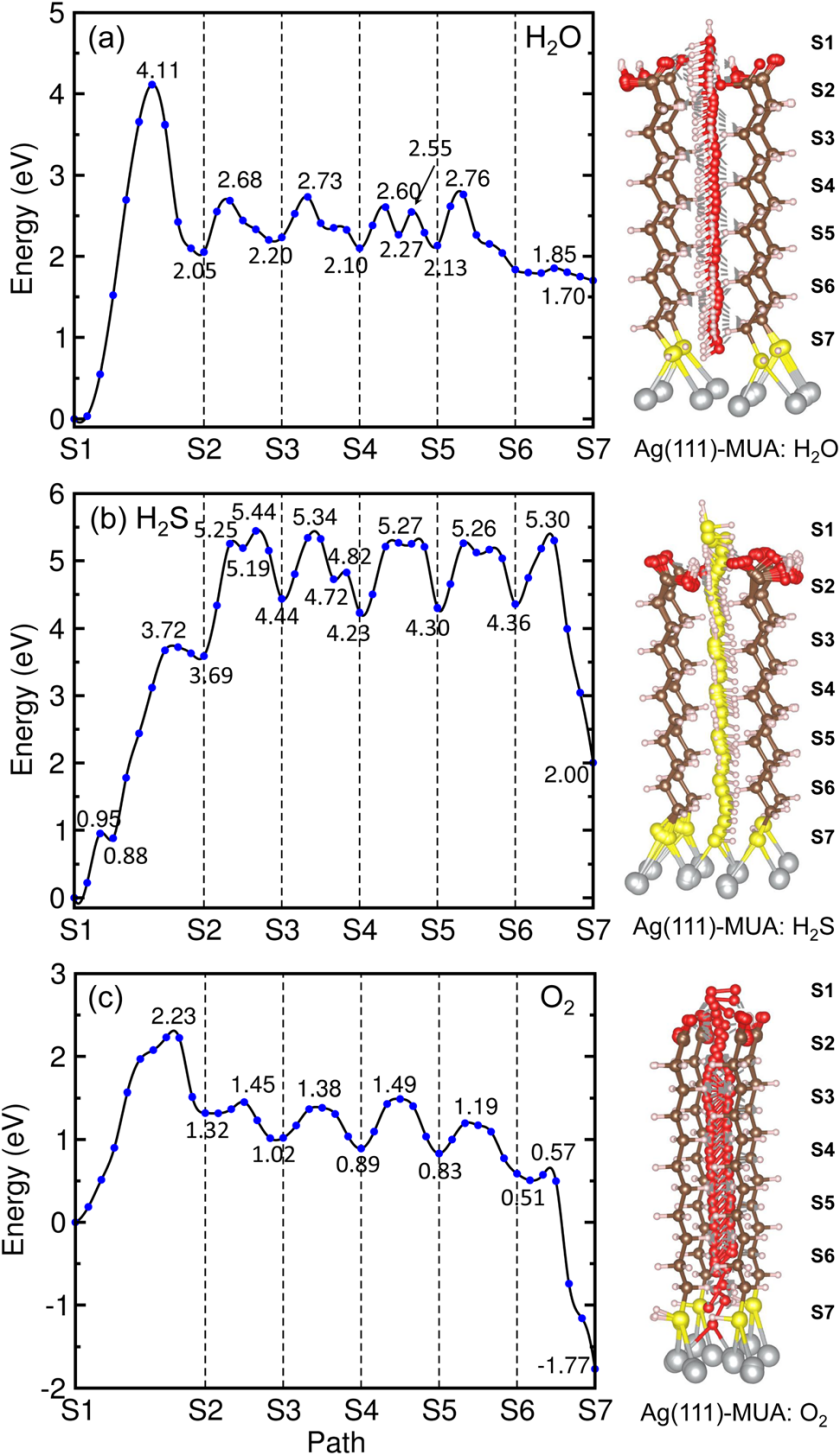
Energy profiles (left panel) for migrations of gas molecules of (a) H_2_O, (b) H_2_S and (c) O_2_ from top of MUA molecule to Ag(1 1 1) surface through space between adjacent MUA molecules, and the corresponding geometries during migration (right panel).

Similar findings were obtained for the migration in the MUA@Ag(1 1 1) complexes as shown in Fig. 9. For the migration of H_2_O molecule, the activation barriers were found to be 4.11, 0.63, 0.53, 0.50, 0.63 and 0.06 eV in the 6 sections (Fig. S12, ESI†). When compared with the Ag(1 0 0) surface, the barrier for insertion is much higher, while other barriers are in the similar oder and the H_2_O molecule is similarly 2.59 Å away from the Ag surface at the final state. For the case of H_2_S migration, the barriers were evaluated to be 3.72, 1.75, 0.90, 1.04, 0.96 and 0.94 eV in the 6 sections (see Fig. S13, ESI†). At the final state, the adsorbate complex of Ag_2_SH_2_ with the Ag–S bond lengths of 2.61 and 2.73 Å was formed like in the case of MUA@Ag(1 0 0) complex. Note that the When compared with the Ag(1 0 0) surface, the first migration barrier is lower, but the following barriers are overall slightly higher. Moreover, the final states were also found to be energetically higher by 1.70 eV for H_2_O and 2.00 eV for H_2_S, respectively.

For the O_2_ migration, the activation barriers were determined to be 2.23, 0.13, 0.36, 0.6, 0.36 and 0.06 eV in the 6 sections as shown in Fig. 9(c). Unlike the Ag(1 0 0) case, the barrier was also found in the first section migration. However, we should note that the barriers for migrations in the following sections are in the same order to the former case. At the final state, we observed an adsorbate complex of Ag_2_O_2_H with the Ag–O bond lengths of 2.32 and 2.35 Å, which is different from the Ag_2_O–Ag_2_O complex formed on the Ag(1 0 0) surface (see Fig. S14, ESI†). The final state at the S7 point was found to be 1.77 eV lower than the initial state at the S1 point, being similar to the Ag(1 0 0) surface. From the calculated activation barriers, the order of difficulty in penetration to the Ag(1 1 1) surface is H_2_O (4.11 eV) → H_2_S (3.72 eV) → O_2_ (2.23 eV), being different from that to the Ag(1 0 0) surface. We note that the reason for being slight different from the Ag(1 0 0) surface might be slight wider interstitial space surround by 4 MUA molecules.

## 4 Conclusions

In this work, we have investigated the adsorption of MUA molecule on Ag(1 0 0) and (1 1 1) surfaces, the adsorption of gas molecules of H_2_O, H_2_S and O_2_ on the MUA@Ag surface complexes, and their penetrations into the Ag surface, using the first-principles calculations with the aim to elucidate the mechanism of corrosion protection of Ag NW electrode. Using the slab supercell models with different sizes of surface cells, we calculated the surface formation energies and identified the surface relaxations, confirming the agreement with the available experimental results. After clarifying the chemical reactivity of the isolated MUA molecule by using the analysis of the Fukui functions and electrostatic potentials, the MUA molecule was suggested to be adsorbed on the Ag surface in the configuration of its vertical arrangement to surface and its SH end contacting with the Ag atoms. The binding energies of the adsorbed MUA molecule to the Ag surface were found to be −0.47 to −1.62 eV for the Ag(1 0 0) surface and −0.52 to −2.06 eV for the Ag(1 1 1) surface, indicating the attraction between the MUA molecule and the Ag surface. From the analysis of optimized geometries and electron density differences, it was found that the Ag–S bonds were newly formed upon the adsorption by the Lewis acid–base reaction. To assess the corrosion resistivity of the MUA monolayer, the adsorption of gas molecules of H_2_O, H_2_S and O_2_ onto the MUA@Ag surface complexes and their penetrations to the Ag surface passing through the interstitial space. The binding energies of gas molecules to the MUA@Ag surface complexes were calculated to be negative, indicating their spontaneous adsorptions. For the migrations of gas molecules, the highest activation barriers were determined to be 2.84, 5.74 and 1.70 eV in the MUA@Ag(1 0 0) and 4.11, 3.72 and 2.23 eV in the MUA@Ag(1 1 1) surfaces for H_2_O, H_2_S and O_2_ molecules, respectively, indicating that the penetrations of H_2_O and H_2_S molecules are much more difficult than that of O_2_ molecule. With these findings, we believe this work can contribute to understanding the mechanism of corrosion protection of Ag NW electrode by passivation with MUA monolayer.

## Author contributions

Chung-Hyok Kim and Yong-Hak Ro developed the original project. Chol-Ryu and Chol-Jun Yu performed the calculations and drafted the first manuscript. Chung-Hyok Kim, Yong-Hak Ro, and Song-Il O assisted with the post-processing of calculation results, and contributed to useful discussions. Chol-Jun Yu supervised the work. All authors reviewed the manuscript.

## Conflicts of interest

There are no conflicts to declare.

## Supplementary Material

RA-013-D3RA06040C-s001
