# Supplementary material for: First-principles study of mercaptoundecanoic acid molecule adsorption and gas molecule penetration onto silver surface: an insight for corrosion protection
Source: RSC Adv. 2023 Oct 25;13(44):31224–33. doi: 10.1039/d3ra06040c (PMC10598515; doi:10.1039/d3ra06040c)
Supplement: RA-013-D3RA06040C-s001 [file RA-013-D3RA06040C-s001.pdf]

Electronic Supplementary Material (ESI) for RSC Advances.
This journal is © The Royal Society of Chemistry 2023

Electronic Supplementary Information – First-principles study of mercaptoundecanoic acid molecule adsorption and gas molecule penetration onto silver surface: An insight for corrosion protection

Chung-Hyok Kim[a], Chol Ryu[b], Yong-Hak Ro[a], Song-Il O[c], Chol-Jun Yu[a*]

[a]*Institute of Electronic Materials, High-Tech and Development Centre, Kim Il Sung University, Pyongyang, PO Box 76, Democratic People's Republic of Korea*

[b]*Computational Materials Design (CMD), Faculty of Materials Science, Kim Il Sung University, Pyongyang, PO Box 76, Democratic People's Republic of Korea*

[c]*Physics Department, O Jung Hub Chongjin University of Education, Chongjin, Hamgyong North Province, Democratic People's Republic of Korea*

Table 1: Bond length and bond angle of MUA molecule determined using different DFT programs including pseudopotential-PAO program SIESTA, pseudopotential plane wave program Quantum ESPRESSO (QE), and density functional tight binding program DFTB.

	SIESTA	QE	DFTB
Bond length (Å)			
S1–H1	1.353	1.355	1.331
S1–C1	1.826	1.828	1.825
C1–H2	1.090	1.103	1.090
C1–H3	1.087	1.104	1.089
C1–C2	1.520	1.525	1.517
C2–H4	1.104	1.107	1.093
C2–H5	1.092	1.105	1.091
C2–C3	1.526	1.530	1.525
C3–H6	1.100	1.107	1.092
C3–H7	1.109	1.107	1.092
C4–H8	1.091	1.104	1.091
C4–H9	1.104	1.105	1.091
C4–C5	1.524	1.527	1.524
C5–H10	1.101	1.107	1.093
C5–H11	1.096	1.105	1.092
C5–C6	1.516	1.513	1.506
C6–O1	1.373	1.366	1.338
C6–O2	1.217	1.216	1.203
O1–H12	0.989	0.988	0.970
Bond angle (deg)			
H1–S1–C1	93.48	96.41	95.82
H2–C1–H3	106.21	106.84	108.97
H4–C2–H5	108.04	105.93	107.78
H6–C3–H7	103.59	105.83	107.62
H8–C4–H9	104.99	105.78	107.54
H10–C5–H11	102.65	104.76	106.75
O1–C6–O2	122.45	122.25	122.05
S1–C1–C2	115.05	114.44	111.99
C1–C2–C3	113.04	112.92	111.23
C4–C5–C6	118.64	117.68	114.41
C5–C6–O2	124.51	124.54	124.53
C5–C6–O1	113.04	113.21	113.42

*Corresponding author: Chol-Jun Yu, Email: cj.yu@ryongnamsan.edu.kp

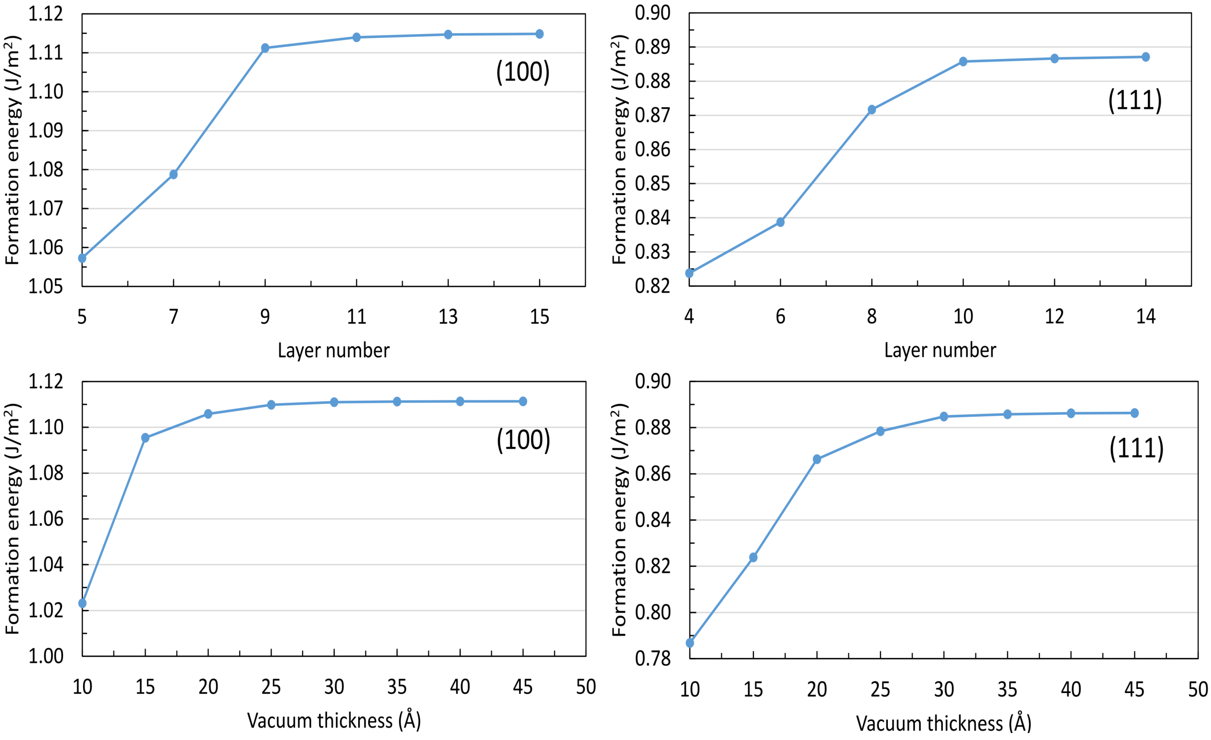

Fig. S1 Convergence test for formation energies of Ag(1 0 0) and (1 1 1) surfaces with respect to the number of layers (top panel) and vacuum thickness (bottom panel).

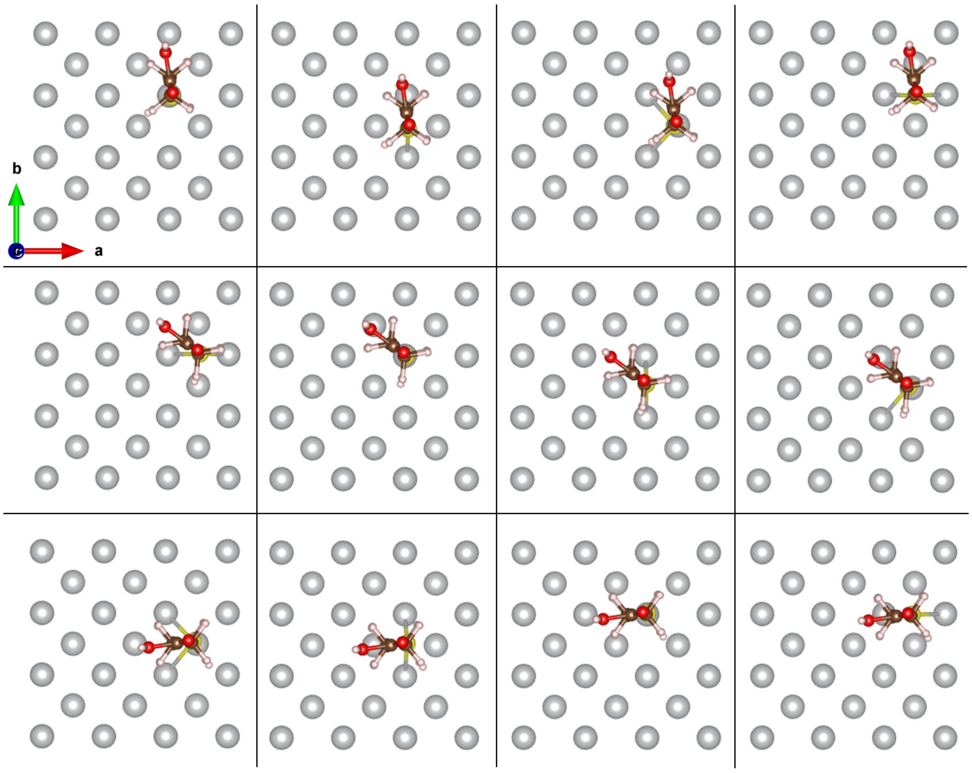

Fig. S2 Different positions of MUA adsorption on Ag(1 0 0) (3 × 3) surface cell.

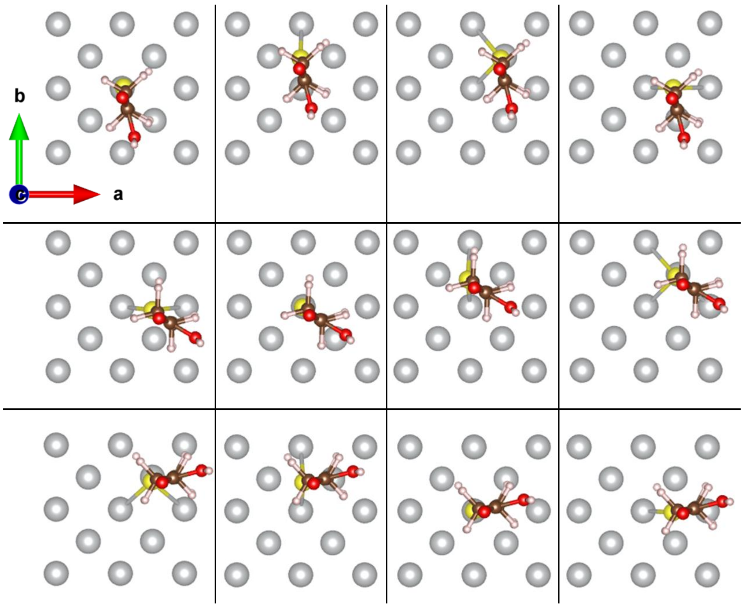

Fig. S3 Different positions of MUA adsorption on Ag(1 0 0) (2 × 2) surface cell.

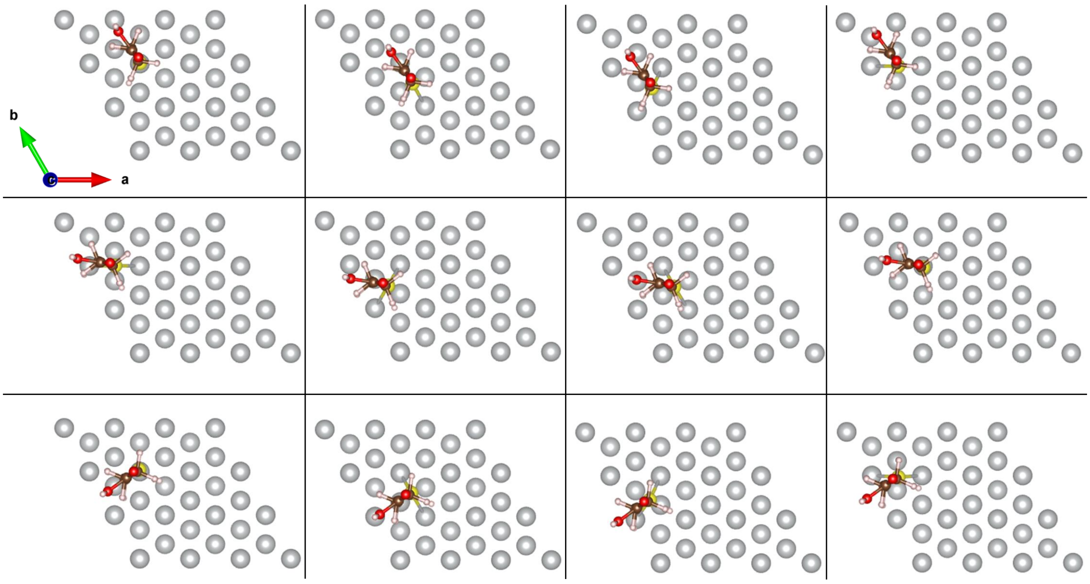

Fig. S4 Different positions of MUA adsorption on Ag(1 1 1) (3 × 3) surface cell.

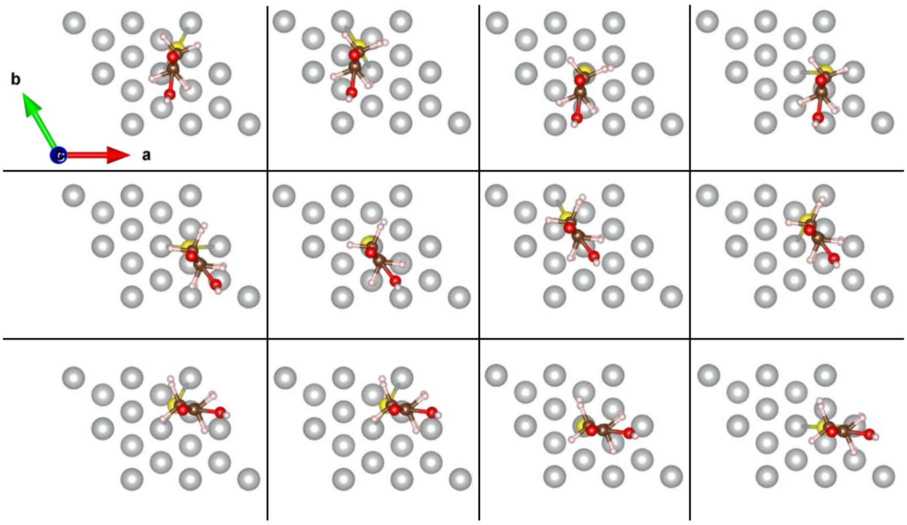

Fig. S5 Different positions of MUA adsorption on Ag(1 1 1) (2 × 2) surface cell.

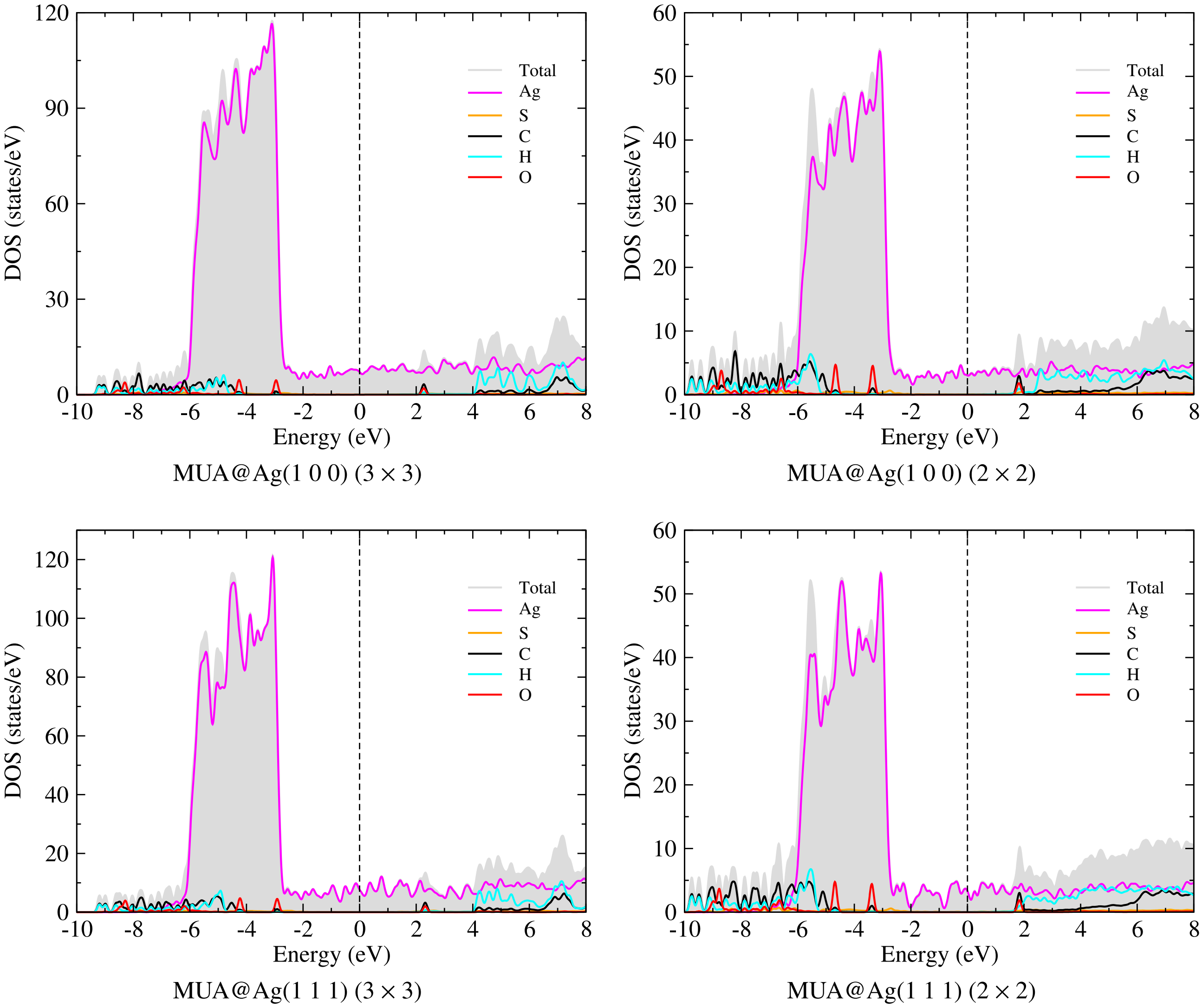

Fig. S6 Atom-projected density of states (DOS) in MUA-adsorbed Ag(1 0 0) and (1 1 1) surface complexes.

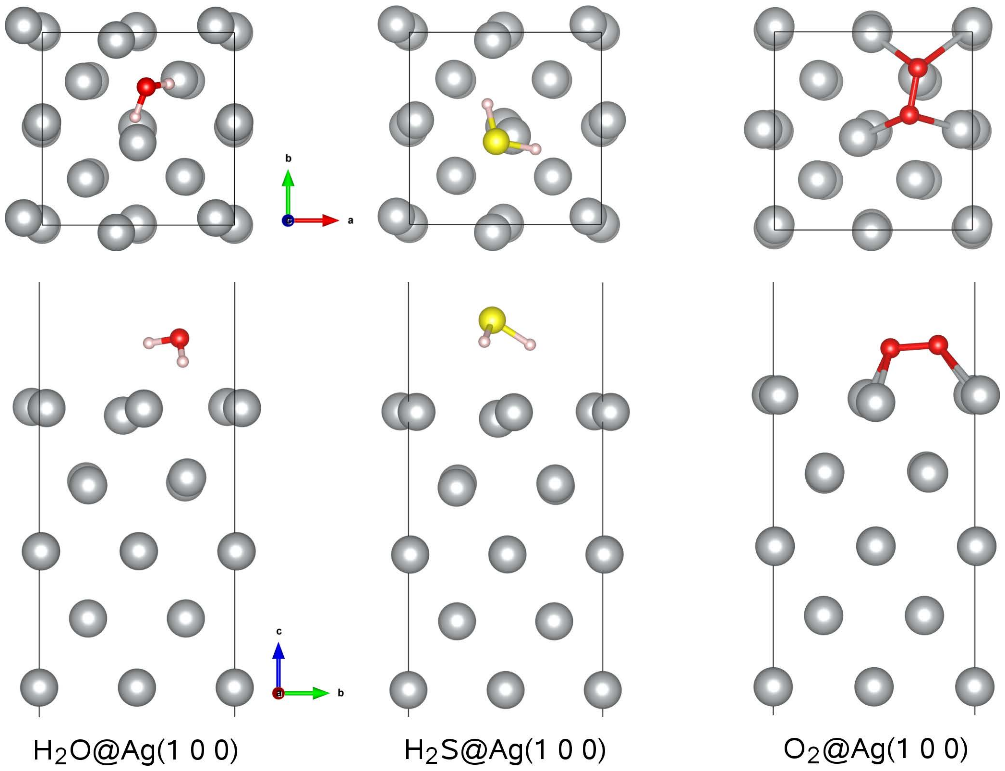

Fig. S7 Optimized geometries of small molecule adsorbed Ag(1 0 0) complexes.

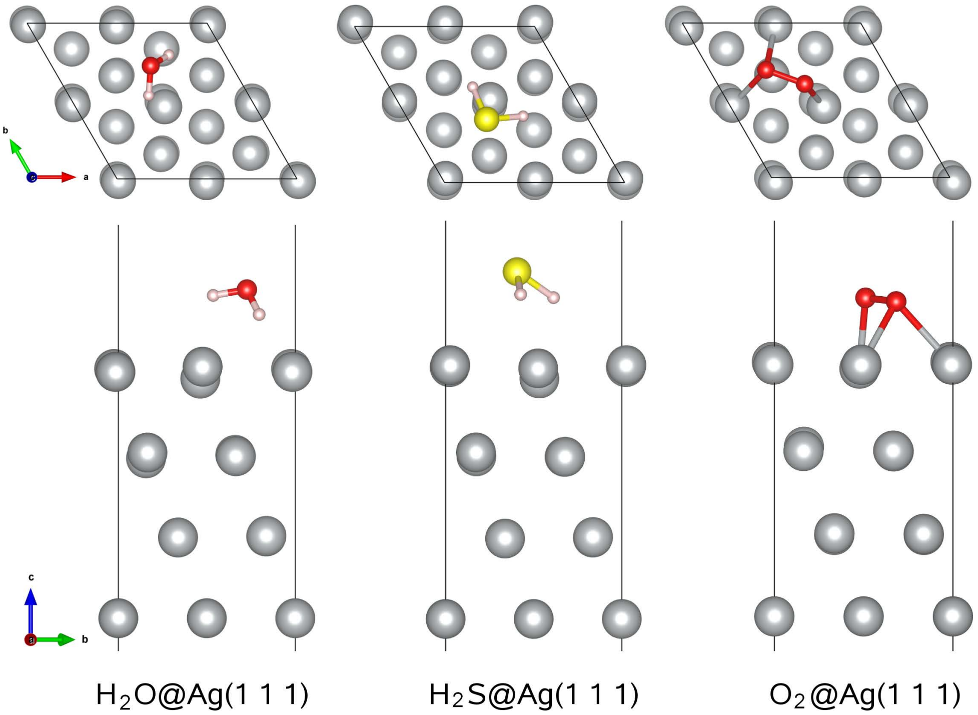

Fig. S8 Optimized geometries of small molecule adsorbed Ag(1 1 1) complexes.

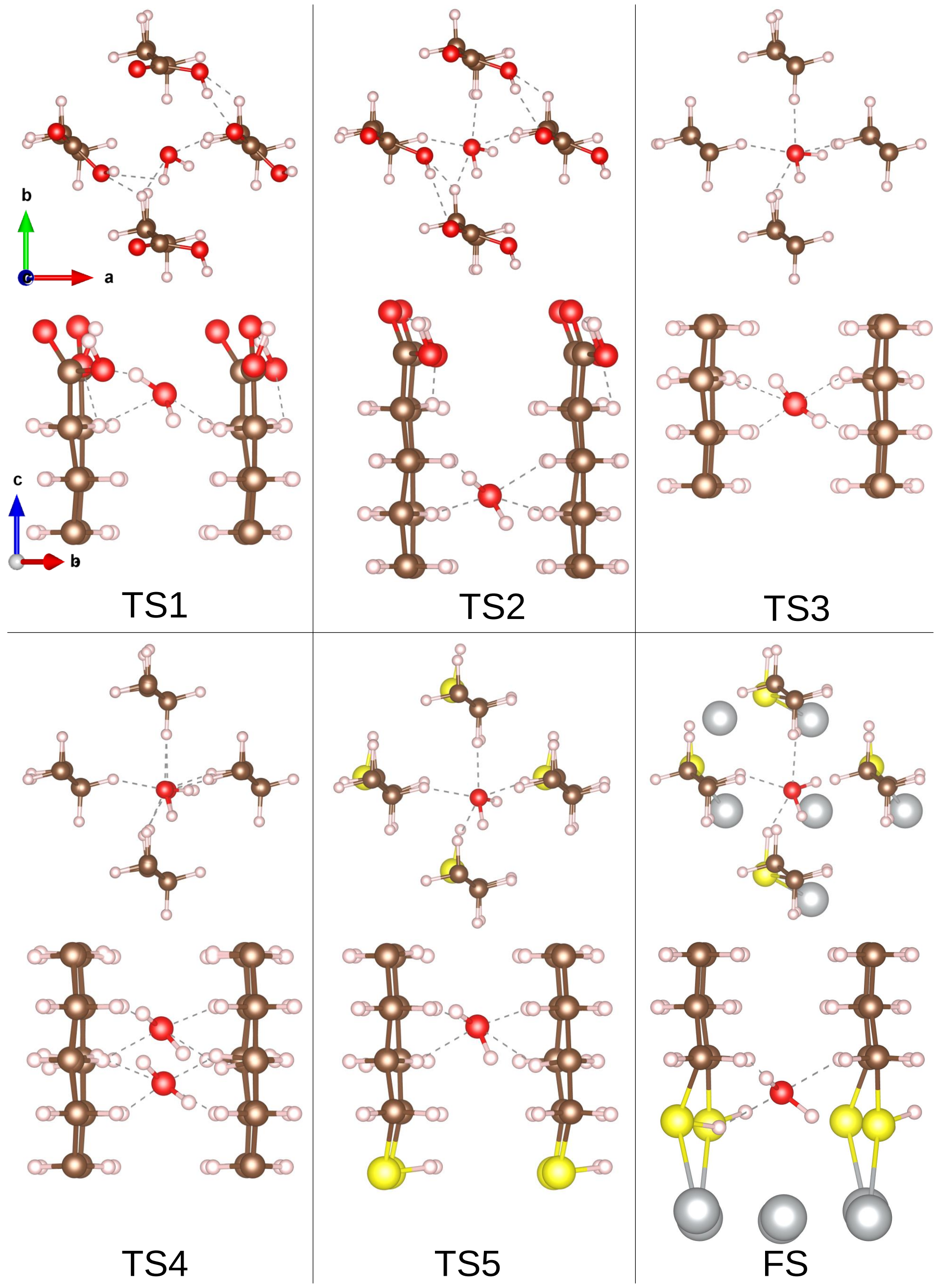

Fig. S9 Geometries of transition states (TS) and final state (FS) for H_2O migration in MUA@Ag(1 0 0) complex.

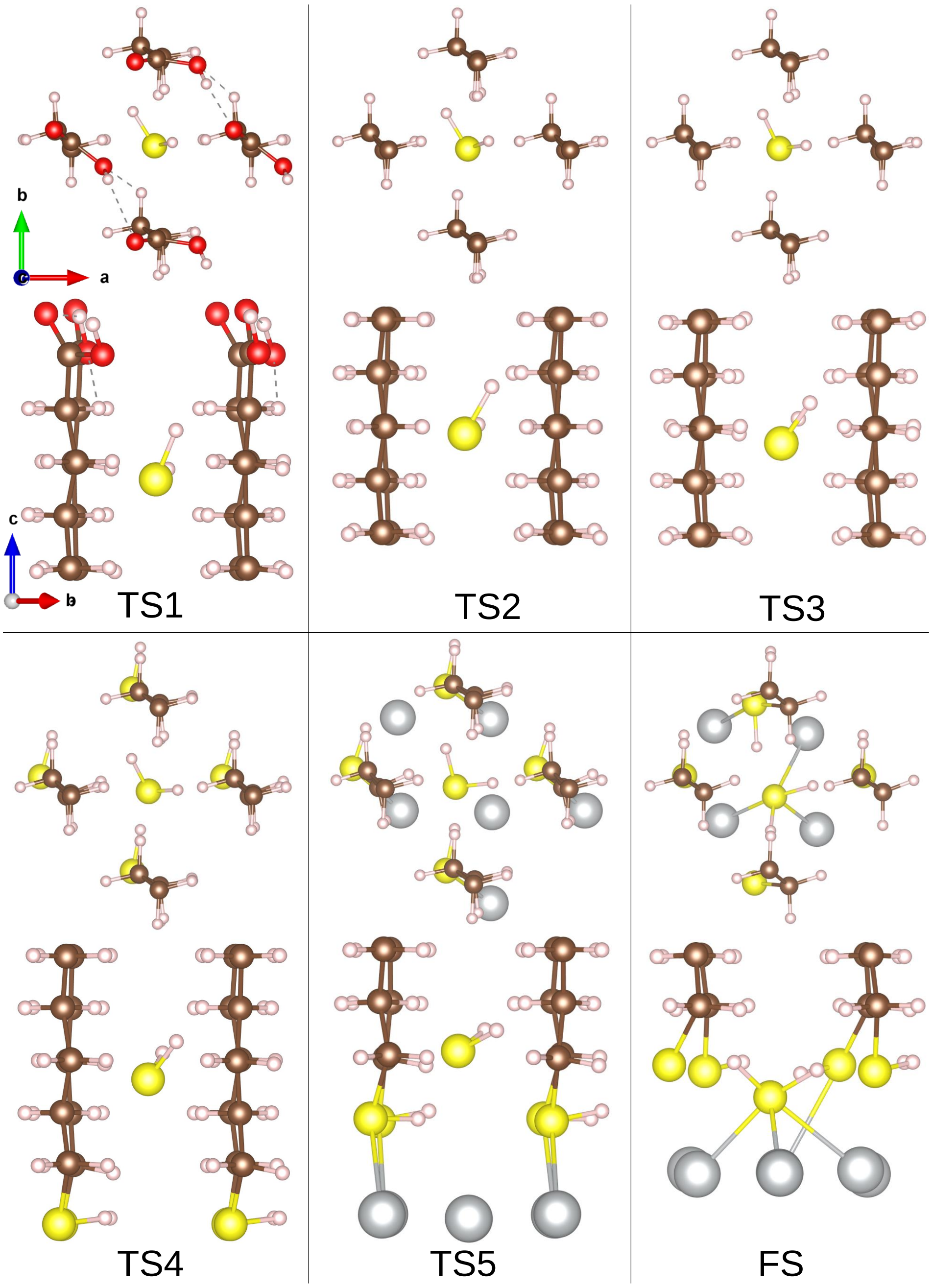

Fig. S10 Geometries of transition states (TS) and final state (FS) for H_2S migration in MUA@Ag(1 0 0) complex.

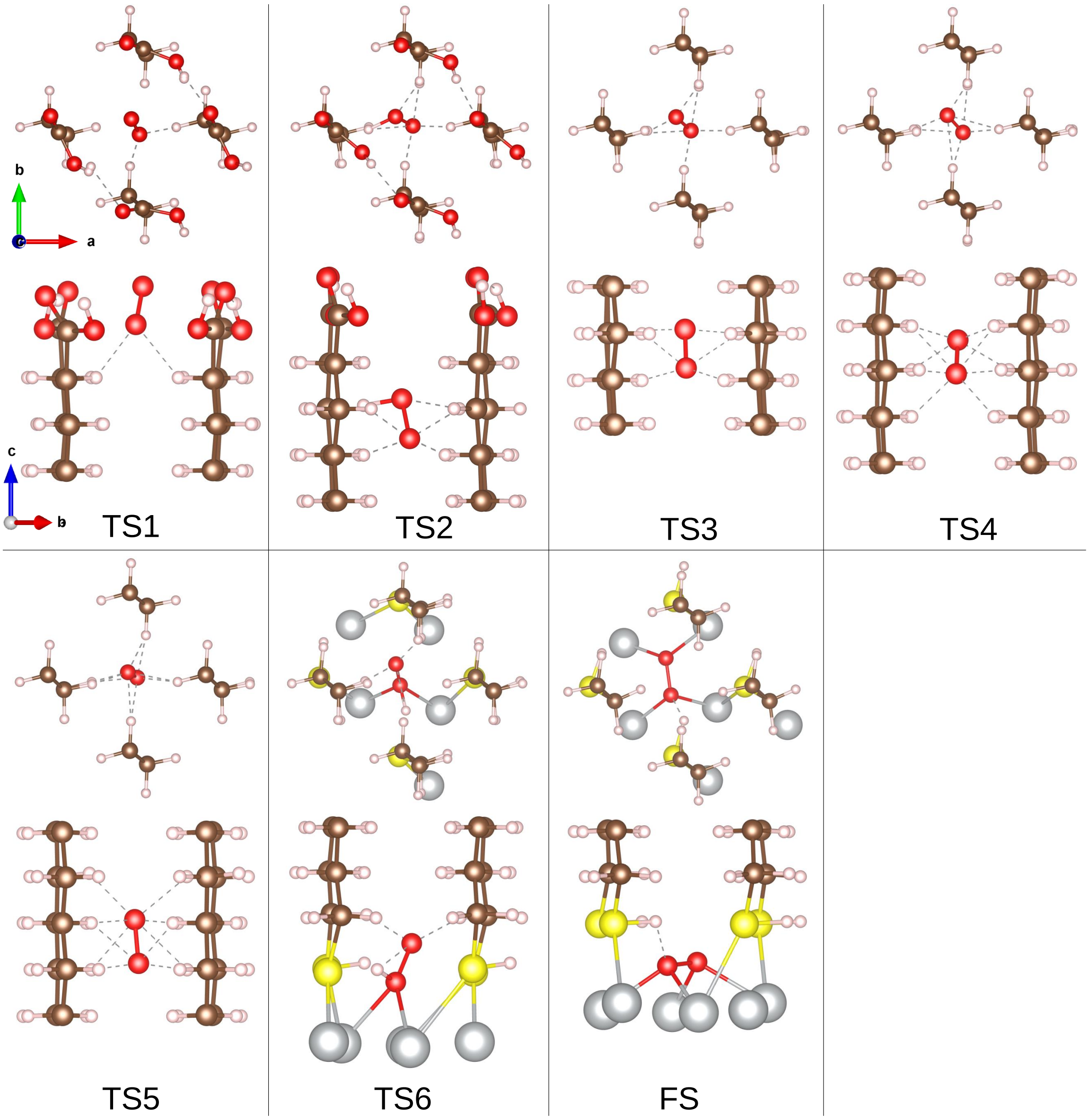

Fig. S11 Geometries of transition states (TS) and final state (FS) for O_2 migration in MUA@Ag(1 0 0) complex.

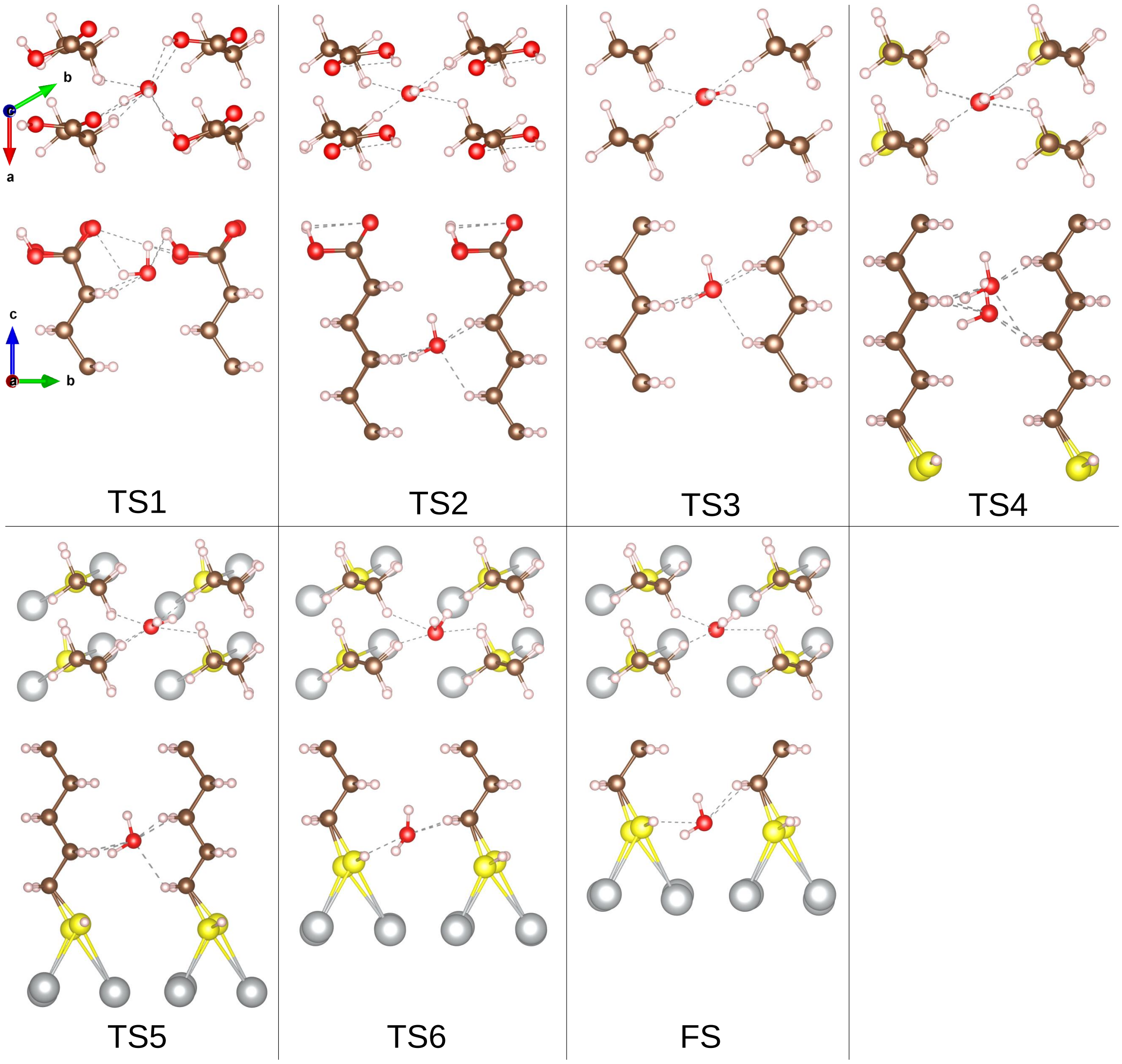

Fig. S12 Geometries of transition states (TS) and final state (FS) for H_2O migration in MUA@Ag(1 1 1) complex.

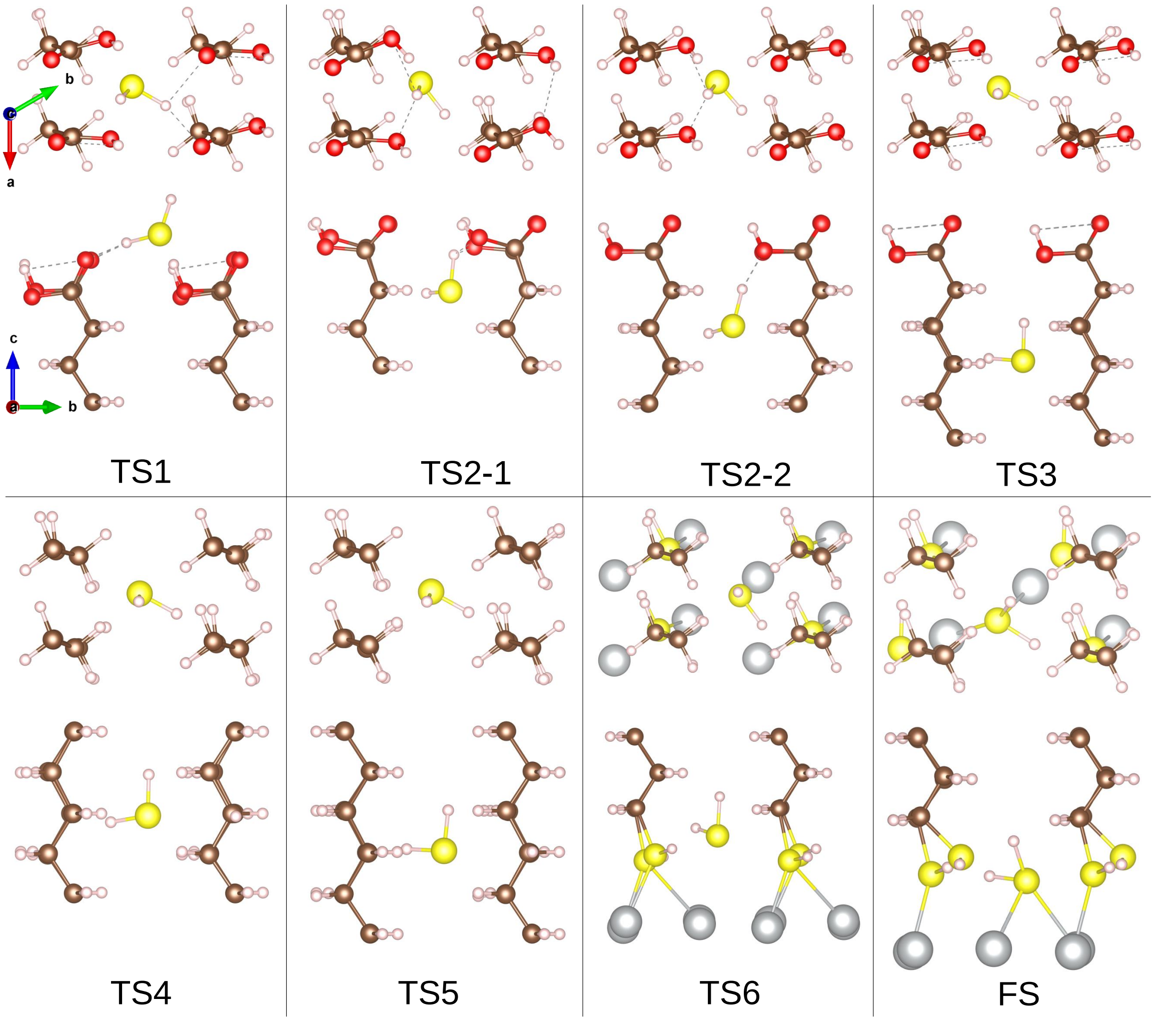

Fig. S13 Geometries of transition states (TS) and final state (FS) for H_2S migration in MUA@Ag(1 1 1) complex.

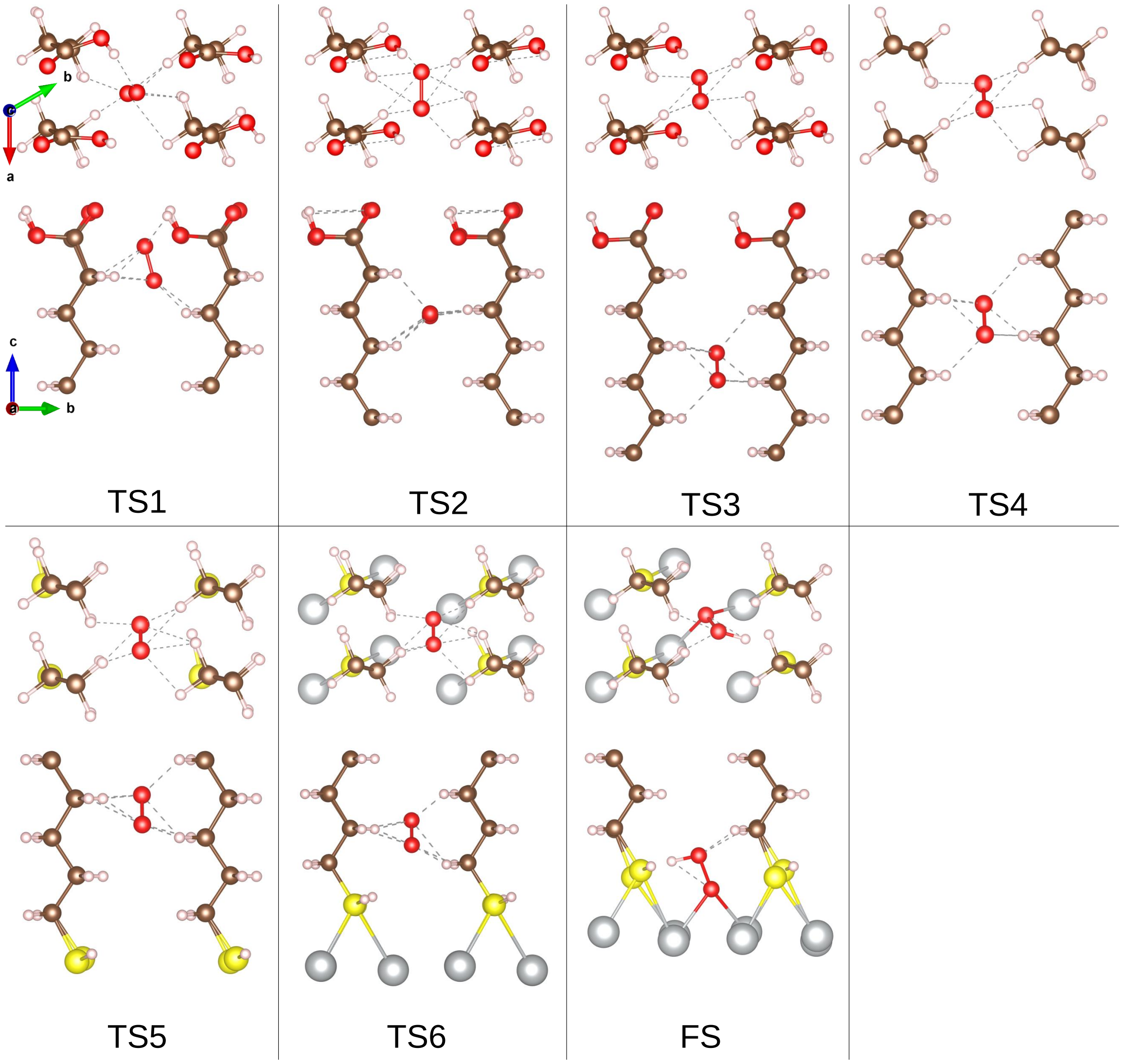

Fig. S14 Geometries of transition states (TS) and final state (FS) for O_2 migration in MUA@Ag(1 1 1) complex.
